# Current Landscape and Future Directions for Mental Health Conversational Agents for Youth: Scoping Review

**DOI:** 10.2196/62758

**Published:** 2025-02-28

**Authors:** Jinkyung Katie Park, Vivek K Singh, Pamela Wisniewski

**Affiliations:** 1 Human-Centered Computing Division School of Computing Clemson University Clemson, SC United States; 2 Department of Library and Information School of Communication and Information Rutgers University New Brunswick, NJ United States; 3 Department of Computer Science School of Engineering Vanderbilt University Nashville, TN United States

**Keywords:** conversational agent, chatbot, mental health, youth, adolescent, scoping review, Preferred Reporting Items for Systematic Reviews and Meta-Analyses, artificial intelligence

## Abstract

**Background:**

Conversational agents (CAs; chatbots) are systems with the ability to interact with users using natural human dialogue. They are increasingly used to support interactive knowledge discovery of sensitive topics such as mental health topics. While much of the research on CAs for mental health has focused on adult populations, the insights from such research may not apply to CAs for youth.

**Objective:**

This study aimed to comprehensively evaluate the state-of-the-art research on mental health CAs for youth.

**Methods:**

Following PRISMA (Preferred Reporting Items for Systematic Reviews and Meta-Analyses) guidelines, we identified 39 peer-reviewed studies specific to mental health CAs designed for youth across 4 databases, including ProQuest, Scopus, Web of Science, and PubMed. We conducted a scoping review of the literature to evaluate the characteristics of research on mental health CAs designed for youth, the design and computational considerations of mental health CAs for youth, and the evaluation outcomes reported in the research on mental health CAs for youth.

**Results:**

We found that many mental health CAs (11/39, 28%) were designed as older peers to provide therapeutic or educational content to promote youth mental well-being. All CAs were designed based on expert knowledge, with a few that incorporated inputs from youth. The technical maturity of CAs was in its infancy, focusing on building prototypes with rule-based models to deliver prewritten content, with limited safety features to respond to imminent risk. Research findings suggest that while youth appreciate the 24/7 availability of friendly or empathetic conversation on sensitive topics with CAs, they found the content provided by CAs to be limited. Finally, we found that most (35/39, 90%) of the reviewed studies did not address the ethical aspects of mental health CAs, while youth were concerned about the privacy and confidentiality of their sensitive conversation data.

**Conclusions:**

Our study highlights the need for researchers to continue to work together to align evidence-based research on mental health CAs for youth with lessons learned on how to best deliver these technologies to youth. Our review brings to light mental health CAs needing further development and evaluation. The new trend of large language model–based CAs can make such technologies more feasible. However, the privacy and safety of the systems should be prioritized. Although preliminary evidence shows positive trends in mental health CAs, long-term evaluative research with larger sample sizes and robust research designs is needed to validate their efficacy. More importantly, collaboration between youth and clinical experts is essential from the early design stages through to the final evaluation to develop safe, effective, and youth-centered mental health chatbots. Finally, best practices for risk mitigation and ethical development of CAs with and for youth are needed to promote their mental well-being.

## Introduction

### Background

Conversational agents (CAs; often called chatbots) are systems with the ability to interact with users using natural human dialogue [1]. Examples of CAs range from customer service chatbots that are available on commercial websites and social media platforms to open-domain, text-based chatbots, such as OpenAI’s GPT-4 and Microsoft’s Bing, and voice assistants, such as Apple’s Siri and Amazon’s Alexa. Driven by advances in the underlying language models, CAs enable 2-way interactive communication with the user and have been applied in multiple domains, including health care [2]. Particularly, CAs are seen as an innovative digital medium to communicate information and resources with younger users, given their high digital literacy and familiarity with chat applications [3]. CAs are now increasingly used by youth for interactive knowledge discovery on sensitive topics, including mental health [4].

Youth are in a unique transitional phase between childhood and adulthood. Following the definition provided by the United Nations, in this review, we use the term *youth* to refer to adolescents and young adults aged between 15 and 24 years. Recent reports show that youth are increasingly experiencing mental health issues these days. For instance, from 2009 to 2019, the proportion of high school students reporting persistent feelings of sadness or hopelessness increased by 40%; between 2007 and 2018, suicide rates among youth aged between 10 and 24 years in the United States increased by 57% [5]. However, they are hesitant to seek professional help on mental health topics due to societal views toward the topics themselves and perceived public stigma and embarrassment associated with help seeking in those topics [6]. With the ability for humanlike interactions with the user, mental health CAs have been developed to support their unique informational, educational, and therapeutic needs related to mental health topics.

Existing systematic reviews on mental health CAs shed light on the potential of CAs to provide relevant information and resources via interactive communication. For instance, mental health CAs are used to deliver prewritten therapeutic and training content for people with depression and autism [7]. A systematic review of 13 studies on the outcome of mental health treatment delivered by mental health CAs found reductions in psychological distress after interacting with the mental health CAs [8]. However, another meta-analysis found conflicting results regarding the effect of chatbots on the severity of anxiety and positive and negative affect [9]. A more recent analysis confirmed that CA-based mental health interventions are effective in improving various mental health conditions in the short term, while substantial long-term effects were not observed [10]; artificial intelligence (AI)–based mental health CAs showed a meaningful reduction in symptoms of depression and distress, with no substantial improvement in overall psychological well-being [11]. Consequently, the trends of the clinical effectiveness of mental health CAs in the existing literature are inconclusive.

A scoping review of 37 studies focused on user perceptions toward mental health CAs showed overall positive opinions toward the CAs such as usefulness and ease of use. At the same time, they found conversations with CAs to be shallow, confusing, or brief [12]. There are some systematic reviews on mental health CAs to support people with specific mental health conditions such as substance use disorder [13], depression and anxiety [14], and serious mental illness [15]. For instance, a systematic review of 7 studies involving CAs for assessing serious mental health illness (eg, major depressive disorder and schizophrenia spectrum disorder) found generally positive outcomes regarding CAs’ diagnostic quality, therapeutic efficacy, and acceptability. However, they revealed a lack of standardized measures for evaluating CAs and insufficient representation of the pediatric population [15]. As such, reviews on mental health CAs are well established and provide important insights into the benefits and pitfalls of existing research on mental health CAs. However, little work has been done to explore the trends in research on mental health CAs for younger populations such as adolescents and youth.

Recently, Balan et al [16] conducted a scoping review of 25 studies on CAs designed to improve the emotional components of mental health (eg, depression and anxiety) of the young population and found that although usability outcomes are optimistic, the clinical effectiveness of CA-based mental health interventions remains inconclusive. While trends in therapeutic CAs to improve the emotions of young populations were studied, a comprehensive trend in research on mental health CAs with various goals (eg, informational and assessment) or social, behavioral, or cognitive aspects of mental health has not been studied in previous work. In addition, trends in design aspects (eg, CA role and characteristics) and evaluation outcomes beyond efficacy (eg, strength and weaknesses of CAs and ethics) were not addressed in previous work. Therefore, to fill the gap in the literature, we conducted a scoping review of 39 studies that focused on CAs to promote the mental health of youth. In this paper, we address the following research questions (RQs):

- RQ1: What are the characteristics of empirical research on mental health CAs designed for youth?
- RQ2: What are the design and computational considerations for mental health CAs for youth?
- RQ3: What are the evaluation outcomes reported in empirical research on mental health CAs for youth?

### Objectives

The objective of this study was to synthesize the current literature on mental health CAs designed for youth to understand the trends in research, the design and computational aspects of the CAs, and the strengths and weaknesses of the current mental health CAs for youth. Following the PRISMA (Preferred Reporting Items for Systematic Reviews and Meta-Analyses) 2020 statement guidelines [17], we conducted a comprehensive review of the existing literature on mental health CAs for youth. We describe this process in the subsequent sections.

## Methods

### Systematic Literature Search Process

We initially searched the literature with the search string (“conversational agent” OR “chatbot”) to explore synonyms of those terms used in the literature. Our initial search informed us of the various alternative terms used to describe CAs or chatbots, which allowed for a more inclusive and thorough search. Our final search string consisted of the following keywords: (“conversational agent” OR “chatbot” OR “virtual agent” OR “virtual assistant” OR “AI assistant” OR “AI bot” OR “social bot”) AND (teen OR adolescent OR youth OR young) AND (mental).

Then, we identified 4 relevant and cross-disciplinary databases that included research on CAs in the health care domain, including ProQuest, Scopus, Web of Science, and PubMed. The same search string was used to retrieve articles across the 4 databases. The searches were limited to journal articles, conference papers, and book chapters written in English. The publication date was not specified. The initial search resulted in retrieving 224 articles from the 4 databases (ie, Web of Science: n=59, ProQuest: n=31, Scopus: n=85, and PubMed: n=49) in February 2024.

### Inclusion and Exclusion Criteria

The purpose of this work was to review mental health CAs designed for youth. Therefore, we included full research papers that (1) were peer reviewed (journal articles and refereed conference proceedings were both included); (2) discussed CAs for providing information or resources or support on mental health topics; (3) discussed CAs designed for youth, adolescents, or young adults; (4) described CAs that permitted 2-way interactions that were fully automated (ie, without human mediation); and (5) included empirical results.

We excluded papers that (1) were nonfull or nonreviewed, such as works in progress, extended abstracts, reports, reviews, and meta-analyses; (2) did not include mental health topics (eg, physical health); (3) included forms of 1-way communication and human-mediated communication; (4) did not consider youth population; (5) did not primarily focus on the CAs (eg, explored CAs as one of the features of the mobile health apps); (6) did not focus on natural human dialogue as a primary communication mode for a 2-way interaction (eg, embodied CAs and facial recognition); and (7) were purely theoretical analyses or a review of existing studies.

### Data Screening

We first removed the duplicate entries from 224 articles. After removing 94 duplicates, we had 130 unique entries. Screening of articles for inclusion was performed in 2 stages. First, we screened the articles by reviewing titles, abstracts, and keywords. Next, we conducted relevancy coding by reviewing full texts based on the abovementioned criteria. The initial screening using titles and abstracts led to the removal of 54 articles. With the 76 remaining articles, we proceeded with the relevancy coding of the full texts. Through this relevancy coding process, 42 articles were removed, and a set of 34 articles were processed for cross-reference. To identify additional relevant papers that were not identified in our initial search, we cross-referenced the citations of 34 articles. Through this cross-reference process, we identified 5 relevant articles to include in our analysis. After 1 more iteration of the cross-reference process, no additional relevant papers were identified, which suggested that we reached a saturation point. The final number of articles that were included in our literature review was 39.

### Data Analysis Approaches

**Figure 1:**
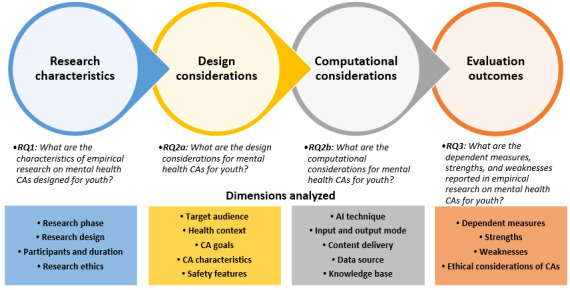
Overview of the analytic framework. AI: artificial intelligence; CA: conversational agent; RQ: research question.

We conducted a thematic analysis to identify major themes and trends in our dataset. We leveraged an iterative approach to our thematic analysis, which involved refining the codes as we gained a deeper understanding of the data. JKP carried out the coding, supported by frequent check-ins with PW for additional expertise on the subject matter. Furthermore, to ensure face validity, VKS quality-checked the coding during the writing of the results. For grounded thematic analysis, we analyzed the papers to identify codes for different dimensions aligning with our RQs. We familiarized ourselves with the literature identified and generated the initial codes. With the initial codes, we coded 20% (8/39) of the dataset and reviewed the codes to ensure that they were representative of our dataset. Once we finalized the codes, we coded the entire dataset. Multiple codes were sometimes assigned to the same paper where necessary. Through the grounded thematic analysis, we identified major dimensions for the characteristics of empirical research on mental health CAs for youth (ie, RQ1), design and computational considerations of mental health CAs for youth (ie, RQ2), and the evaluation outcomes of empirical research on mental health CAs for youth (ie, RQ3). Figure 1 is an overview of the framework, including RQs, dimensions, and codes that were analyzed in this review to understand the trends in research on mental health CAs for youth.

## Results

### Overview

**Figure 2:**
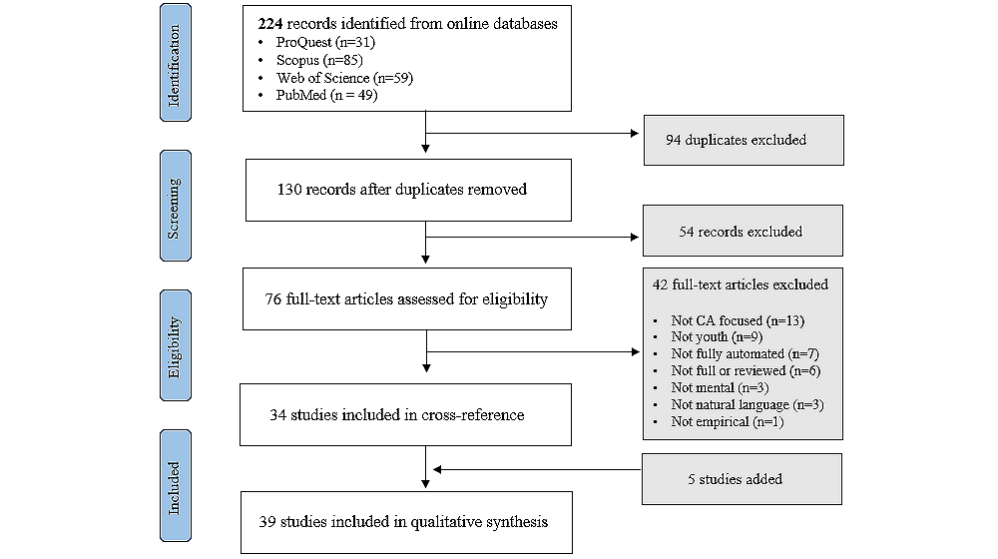
PRISMA (Preferred Reporting Items for Systematic Reviews and Meta-Analyses) flowchart for the study.

Overall, we included 39 studies in this scoping review [4,18-55]. Figure 2 presents the data screening process following the PRISMA guidelines (see Multimedia Appendix 1 for the PRISMA Extension for Scoping Reviews checklist). The reviewed studies were published between 2011 and February 2024. Most of the studies (28/39, 72%) were published between 2021 and 2024, with a few published before 2021 (11/39, 28%).

The reviewed studies were conducted in various regions, including the United States (8/39, 20%); New Zealand (5/39, 13%); Australia (4/39, 10%); the United Kingdom, China, and Brazil (3/39, 8% each); Germany, Italy, and Norway (2/39, 5% each); and Argentina, Bangladesh, Belgium, Canada, the Netherlands, the Philippines, and Portugal (1/39, 3% each).

### Characteristics of Empirical Research on Mental Health CAs for Youth

#### Most of the Empirical Research Involved Development and Summative Evaluation; Less Work Involved Formative Evaluation to Redesign Mental Health CAs With and for Youth

Most of the reviewed studies (36/39, 92%) included summative evaluation, followed by development (29/39, 74%), design (15/39, 38%), and formative evaluation (15/39, 38%; Table 1 and Multimedia Appendix 2 [4,18-55]). All empirical research included an evaluative phase to assess the mental health CAs for youth. Most of the studies included summative evaluation (36/39, 92%) and formative evaluation (15/39, 38%). In 31% (12/39) of the studies, both formative and summative evaluations were conducted. The term formative and summative evaluations originally referred to methodological frameworks for evaluating instructional materials [56]. In human-computer interaction research, summative evaluation is the evaluation of a design after it is nearly complete, often used during field testing. Formative evaluation is conducted during user interface development, often iteratively to achieve the desired usability level [57].

In the formative evaluation work that we reviewed, researchers interacted with youth populations to gain early feedback on the initial CA design through interviews, focus groups, and design workshops [31,32,49]. In most of the studies that included formative evaluation (12/15, 80%), summative evaluations were followed after the iterative design process. For instance, Ludin et al [33] conducted a formative user testing with school guidance counselors, clinicians, and youth to learn about the issues youth faced during the COVID-19 pandemic and lockdowns. The youth’s opinions from the formative evaluations were used to inform the ongoing content iteration of the prototypes. Once the prototype was developed, the authors conducted a summative user testing to assess the usability and acceptability of the chatbot with 127 youth participants [33].

**Table 1:** Characteristics of the empirical research on mental health CAs^a^ for youth (N=39).

Dimensions and codes				Value, n (%)		Key trends
**Research phase**						Most of the empirical research involved development and summative evaluation; less work involved formative evaluation to (re)design mental health CAs with youth
	Design		15 (38)			
	Development		29 (74)			
	Formative evaluation		15 (38)			
	Summative evaluation		36 (92)			
**Research design**						Most of the research involved user testing or experiments to evaluate mental health CAs
	User testing		18 (46)			
	Experiment		18 (46)			
**Research participants and duration**						Most commonly, the research on mental health CAs was conducted with <100 participants who are adolescents and young adults without mental health conditions for <4 wk
	**Participant age (y)**					
		Adolescents (12-18)	16 (41)			
		Young adults (18-30)	14 (36)			
		Youth (14-25)	9 (23)			
	**Participant group**					
		General population	21 (54)			
		At-risk population	18 (46)			
	**Number of participants**					
		<100	28 (72)			
		100-200	6 (15)			
		>200	2 (5)			
	**Duration of CA interaction**					
		1 time	6 (15)			
		<4 wk	12 (31)			
		4-8 wk	5 (13)			
		9-16 wk	1 (3)			
	**Research ethics**					Research ethics such as institutional review and consent were addressed in most research; however, only a few research studies addressed data privacy and safety of youth participants
		Consent	26 (67)			
		Institutional review	26 (67)			
		Data confidentiality	8 (20)			
		Safety	4 (10)			

^a^CA: conversational agent.

#### Research on Mental Health CAs Was Conducted With a Small Numbers of Adolescents and Young Adults Without Mental Health Conditions for <4 Weeks

##### Participant Group

Most of the participants in the reviewed studies were adolescents (16/39, 41%) aged between 12 and 18 years and young adults (14/39, 36%) aged >18 years. In fewer studies, the participants were youth (9/39, 23%) aged between 14 and 25 years. Overall, in a slightly higher proportion of the empirical studies, the participants were general youth (21/39, 54%) compared to at-risk youth (18/39, 46%), such as those who are experiencing depression [51], anxiety [18], body image concerns [23], and alcohol abuse [20].

##### The Number of Participants

The number of youth participants ranged from a minimum of 3 young adults with autism to explore the acceptability of cognitive behavioral therapy (CBT)–based mental health CA through in-depth interviews [53] to a maximum of 798 adolescents to evaluate the effectiveness and user engagement through a survey [48]. In most of the research, the number of participants was <100 (28/39, 72%), with few studies having participants between 100 and 200 (6/39, 15%) and >200 (2/39, 5%).

##### Duration of CA Interaction

Youth participants interacted with mental health CAs for a minimum of 30 minutes [20,25] to a maximum of 16 weeks [47]. In most of the reviewed studies, participants interacted with CAs for <4 weeks (12/39, 31%) or once (6/39, 15%). In fewer studies, participants interacted with CAs for 4 to 8 weeks (5/39, 13%) or 9 to 16 weeks (1/39, 3%). In general, short-term engagement with CAs was for user testing to assess user experience and acceptability. The long-term engagement with CAs was for the experiments to assess the effectiveness of CAs in reducing mental health conditions.

#### Most of the Research Involved User Testing or Experiments to Evaluate Mental Health CAs

##### User Testing

The most prevalent research design used in the empirical studies was user testing (18/39, 46%) to assess user engagement and user experience of the CAs. For instance, in a study by Beilharz et al [23], 17 adolescents and 8 parents or caregivers in Australia participated in focus group interviews to evaluate the acceptability, ease of use, and design of the *KIT* prototype, a CA designed to support people with concerns about body image and eating issues. In another study, Gabrielli et al [37] conducted a user test with 21 adolescents over 4 weeks in Italy to assess the user experience and perceived value of content delivered by mental health CAs. Surveys with Likert scale measures and open-ended questions were used to evaluate overall usefulness, ease of use, the value of the content, and suggestions for improvement [37].

##### Experiment

Another prevalent research design was an experiment (18/39, 46%), which was conducted to explore the effectiveness of CAs in reducing mental health conditions. With the randomized controlled trial design, for instance, young adults who completed active cancer treatment in 5 years were randomly assigned to either immediate access to mental health CA (ie, experimental group) or access to only daily emotion ratings and access to full chatbot content after 4 weeks (ie, control group). After 4 weeks, participants in the experimental group reported an average reduction in anxiety, while the control group reported an increase in anxiety [43]. With noncontrolled experiments, for instance, 105 young adults interacted with the same mental health CAs for 15 days and completed the surveys asking about their mental health conditions (ie, Patient Health Questionnaire [PHQ]) before and after the 15-day intervention period. The comparison of the average survey scores before and after the intervention confirmed that the overall scores decreased after interacting with the mental health chatbot [36].

#### Research Ethics Such as Institutional Review and Consent Were Addressed in the Majority of Research; However, Only a Few Research Considered Data Privacy and Safety of Youth Participants

In most of the reviewed studies, authors explicitly stated that they acquired participant consent (26/39, 67%) and that the studies were approved by their institutional review board (26/39, 67%). Beyond institutional review and participant consent, which are mandatory in many institutions, in some studies, authors addressed considerations for privacy or confidentiality of youth’s digital trace data related to mental health (8/39, 20%). In only a small portion of the reviewed studies (4/39, 10%), the authors provided support to promote the safety of youth participants in empirical research in the mental health context. For instance, in the study by Nicol et al [22], there was safety monitoring provided by the study team by tracking the digital PHQ-9 assessment over 12 weeks. When the PHQ-9 assessment results indicated suicidal ideation, the principal investigator contacted the participant’s primary care providers to discuss the next steps regarding the assessment of suicide risk. By the end of the 12-week study, 56% (10/18) of the participants triggered at least 1 alarm to assess for suicidal ideation [22]. In another study by Liu et al [47], participants were informed that professionals would intervene by telephone (ie, mental assistance hotline) when the participants reported that they needed emergency psychological assistance. In 10 papers, research ethics were not mentioned. None of the reviewed papers mentioned risk mitigation plans and mandated reporting or provided critical reflections on best practices for working with youth in the mental health context.

### Design Considerations of Mental Health CAs for Youth

#### Most of the Mental Health CAs Were Designed for Older and General Youth Populations

In most of the studies, the target audience of the CAs were youth or young adults (22/39, 56%), adolescents (12/39, 31%), and college students (6/39, 15%). In most of the studies (27/39, 69%), CAs were designed for general youth populations. In a small proportion of the reviewed studies (12/39, 31%), the CAs were designed for at-risk youth populations (Table 2 and Multimedia Appendix 3 [4,18-55]).

**Table 2:** Design considerations of mental health CAs^a^ for youth (N=39).

Dimensions and codes				Value, n (%)		Key trends
**Target audience**						Most of the mental health CAs were designed for older and general youth populations
	**Age group**					
		Youth or young adults	22 (56)			
		Adolescent	12 (31)			
		College students	6 (15)			
	**Health condition**					
		General youth	27 (69)			
		At-risk youth	12 (31)			
**CA goals and health context**						Most mental health CAs for youth are designed to provide Educational or informational support to promote the general mental well-being therapeutic support to alleviate mental health conditions such as depression, anxiety, and stress
	**Health context**					
		Mental well-being	15 (38)			
		Depression	12 (31)			
		Anxiety	9 (23)			
		Stress	8 (20)			
		Substance use	3 (8)			
		Body image	2 (5)			
		Phone addiction	1 (3)			
	**CA goals**					
		Treatment	25 (64)			
		Education or training	22 (56)			
		Informational	15 (38)			
		Assessment	10 (26)			
		Monitoring	2 (5)			
		Behavioral change	2 (5)			
	**CA role and characteristic**					Most mental health CAs for youth were designed to be life coaches or older peer mentors in friendly and empathetic tones with few options to personalize agent characters
	**CA role**					
		Coach or peer	11 (28)			
		Health care professional	7 (18)			
	**CA characteristics**					
		Friendly	16 (41)			
		Empathetic	11 (28)			
		Culture specific	4 (10)			
		Gender specific	3 (8)			
		Simple and factual	2 (5)			
	**Personalization**					
		CA character	5 (13)			
		App appearance	1 (3)			
		User avatar	1 (3)			
	**Safety features**					Most of the mental health CAs did not have the safety features in place
		Reminder	12 (31)			
		Emergency contact	10 (26)			
		Alert to adults or experts	2 (5)			

^a^CA: conversational agent.

**Figure 3:**
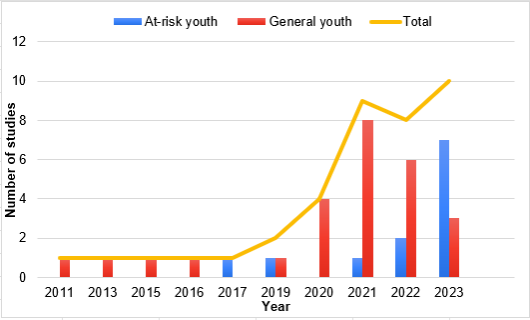
Target audience of the mental health conversational agents (CAs) by year (n=38).

Within those 12 studies, 5 (41%) studies focused on designing mental health CAs for young adults with depressive symptoms [18,19,29,51,52]. Few studies focused on CAs to promote the mental health of adolescents with type 1 diabetes [26]; adolescents with body image and eating issues [23]; young adults being treated for cancer [43]; youth at risk of HIV and sexually transmitted infections [30]; young adults from immigrant and refugee communities [49]; lesbian, gay, bisexual, transgender, queer, or questioning (LGBTQ+) youth [50]; and young adults with autism [53]. There was a trend in which the number of mental health CAs designed for at-risk youth increased, while the number of CAs for general youth decreased (Figure 3). Overall, most of the mental health CAs were designed for older (22/39, 56%) or general (27/39, 69%) youth populations, with the current trend toward designing CAs for youth who are at risk of mental health conditions.

#### Mental Health CAs Are Designed to Promote the General Mental Well-Being of Youth as Well as Help Alleviate Mental Health Symptoms of Depression, Anxiety, and Stress

##### Health Context

Within the studies that focused on mental health CAs for youth, most of the CAs were designed to promote the general mental health or well-being of youth (15/39, 38%; eg, by promoting life skills and resilience), followed by those aimed to reduce depression (12/39, 31%), anxiety (9/39, 23%), and stress (8/39, 20%). There are a few papers that focused on mental health CAs to address the issues of substance use (3/39, 8%; ie, alcohol and drug abuse) [20,28,42], body image (2/39, 5%) [23,48], and phone addiction (1/39, 3%) [21]. Overall, nearly half (19/39, 48%) of the existing research on mental health CAs focused on supporting youth with symptoms of depression, anxiety, and stress.

##### CA Goals

Most CAs (25/39, 64%) for mental health were designed to help alleviate mental health symptoms such as depression and anxiety, followed by educational or training content (22/39, 56%) to promote mental health and informational support on mental health topics (15/39, 38%). Some mental health CAs were designed to assess mental health symptoms (10/39, 26%) and monitor mental health conditions (2/39, 5%). Two mental health CAs were designed to promote behavior changes such as reduced screen time [21] and reduced alcohol consumption [41]. Overall, in most of the work, the CAs were designed to help alleviate youth’s mental health symptoms or to provide educational intervention to promote youth’s mental well-being, while less work has been done on CAs for informational support or the assessment or monitoring of youth’s mental health condition.

#### Mental Health CAs Were Designed to Be Life Coaches or Older Peer Mentors With Friendly and Empathetic Tones, With Few Options to Personalize Agent Characters

##### CA Role

In 51% (20/39) of the studies, the CA role was mentioned. In many of those studies (11/39, 28%), the mental health CAs were designed as older peers or younger coaches to guide youth with therapeutic modules and provide youth with information to promote mental health. For instance, a mental health CA was designed to be a young person who messages the user once a day. The interaction was designed to be a brief conversation with a friend who checks in and has a helpful tip or an anecdotal story to share to help reduce stress [38]. In other studies, mental health CAs were designed to provide formal health professional–like support (7/39, 18%), for instance, that emulates therapists who encourage patients to explore their problems by asking questions to provide personalized mental health support [52].

##### CA Characteristics

Meanwhile, most mental health CAs were designed to provide friendly (16/39, 41%) or empathetic (11/39, 28%) responses. For instance, in response to loneliness reflected in users’ input, the chatbot replied, “I’m so sorry you’re feeling lonely. I guess we all feel a little lonely sometimes,” or if participants showed excitement in their input, the chatbot replied, “Yay, always good to hear that!” [18]. In 10% (4/39) of the studies, mental health CAs were designed to be culture specific. For instance, in a study by Ludin et al [33], researchers collaborated with groups of Indigenous and non-Indigenous people (ie, Māori and Pākehā) in New Zealand to cocreate mental health CA called “Aroha,” meaning “caring and kind” in their native language. The mental health strategies and activities were designed for and targeted Māori youth and their families [33]. A few mental health CAs were gender specific (3/39, 8%) or designed to provide short and simple answers (2/39, 5%).

##### CA Characters and Avatars

In most of the studies (33/39, 85%), options to personalize mental health CA characters were not addressed. In a few studies (6/39, 15%), features to support personalization of CA characters were discussed. For instance, for the CA to promote youth resilience, the avatars were designed as older peers with users’ choice of gender and ethnicity representative of the target population [32]. In another study, the authors designed the body image CA with an option to choose between male and female versions of the CA avatar [48]. Some studies designed mental health CAs in which users can customize their avatars [52] and app appearance [53]. In 1 study, youth preferred to choose from 3 to 4 characters with variations of gender, avatar, age, and social role (ie, health professional vs younger coach-like) instead of personalizing each aspect separately [19].

#### The Majority of the Mental Health CAs Did Not Have the Safety Features in Place

Most of the studies (21/39, 54%) did not address the safety features of the designed mental health CAs. In 46% (18/39) of the studies, safety features were discussed, including a reminder that users are interacting with chatbots, not human experts, and chatbots are not replacements for health care providers or places for seeking help (12/39, 31%). There were safety features to provide emergency contact (10/39, 26%), such as crisis hotlines and those that refer to health professionals. In 2 studies, alert features were implemented to notify a trusting adult or providers when a youth was identified as a risk to themselves or others [22,31]. For instance, if trigger words such as self-harm, suicide, death, dead, kill, or die were used by youth, the alert feature to notify adults was activated. The alert recipient is a primary support contact who was identified and confirmed during the initial consent process [31]. Overall, safety features of mental health CAs were discussed in less than half of the reviewed articles (21/39, 54%).

### Computational Considerations of Mental Health CAs for Youth

#### Most of the Mental Health CAs Were Prototypes Built Upon Existing Mobile Chat Apps

##### Maturity of the Device

In most studies (26/39, 67%), mental health CAs were prototypes. In 18% (7/39) of the studies, CAs were developed as fully functioning systems, and in 20% (8/39) of the studies, researchers evaluated existing systems developed in other studies. In 1 study [24], CAs were explored as concepts without having actual systems (Table 3 and Multimedia Appendix 4 [4,18-55]).

**Table 3:** Computational considerations of mental health CAs^a^ for youth (N=39).

Dimensions and codes				Value, n (%)		Key trends
**System characteristics**						Most of the mental health CAs were prototypes built upon existing mobile chat apps
	**Maturity of device**					
		Prototype	26 (67)			
		Fully functioning system	7 (18)			
		Existing system	8 (20)			
		Concept	1 (3)			
	**Delivery channel**					
		Mobile	16 (41)			
		Web	11 (28)			
		Desktop	6 (15)			
**Communication mode**						All CAs supported text-based input (free text along with quick options) with a few that supported voice input; all supported textual output, with many of them supporting visualized output such as image and video
	**Input mode**					
		Text	39 (100)			
		Text+speech	2 (5)			
		Free text+options	19 (49)			
		Free text	13 (33)			
		Quick options	5 (13)			
	**Output mode**					
		Text	39 (100)			
		Image	21 (54)			
		Video	12 (31)			
		Audio	7 (18)			
		Game	6 (15)			
	**AI^b^ technique**					Free-textual inputs were processed via NLP^c^, while prewritten content was delivered via rule-based programming
		NLP	19 (49)			
		Rule based	15 (38)			
	**Content delivery**					Most mental health content was delivered in flexible ways; in some cases, prewritten content was delivered in a structured manner
		Flexible	25 (64)			
		Structured	8 (20)			
		Semistructured	3 (8)			
	**Knowledge base**					Mental health content was built upon evidence-based expert knowledge of cognitive and behavioral therapy and positive psychology
		CBT^d^	19 (49)			
		Positive psychology	7 (18)			
		Other therapeutic content	7 (18)			
		Clinical expert knowledge	6 (15)			

^a^CA: conversational agent.

^b^AI: artificial intelligence.

^c^NLP: natural language processing.

^d^CBT: cognitive behavioral therapy.

##### Delivery Channel

Mental health CAs were delivered via diverse channels. Most CAs were delivered through mobile apps (16/39, 41%), followed by web applications (11/39, 28%) and desktop applications (6/39, 15%). In 28% (11/39) of the studies, CAs were made available on >1 channel. In many studies (16/39, 41%), CAs were delivered using existing chat applications such as Facebook Messenger (eg, [32,33,36]), WhatsApp [51], Windows Live Messenger [42], WeChat [47], and Telegram [27].

#### All CAs Supported Text-Based Input; Most CAs Supported Multimedia Output

##### Input Modality

**Figure 4:**
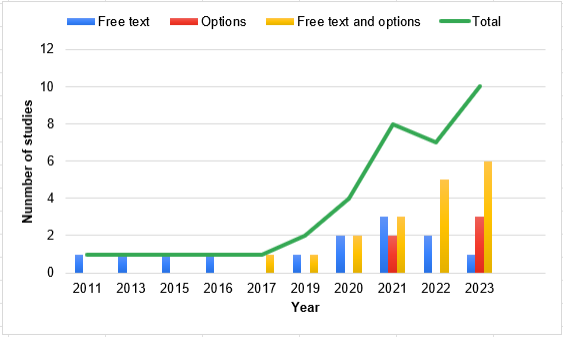
Input mode of the mental health conversational agents (CAs) by year (n=37).

In all the reviewed studies (39/39, 100%), mental health CAs supported text as the primary mode of input. In 5% (2/39) of the studies, mental health CAs supported audio input along with textual input [47,51]. For text-based CAs, by extracting keywords related to mental health such as depression and anxiety from user inputs, CAs extract necessary features to develop different classification models or assess their sentiment [46,52]. For voice-based CAs, the key components include the use of automatic speech recognition and natural language understanding to comprehend users’ input [47]. Text-based and voice-based chatbots are similar in their design; however, text-based CAs do not include automatic speech recognition components and instead rely on text as the primary modality of input and output. In most of the reviewed studies, CAs supported free text along with quick options (eg, “yes” or “no”) as user input (19/39, 49%), rather than free text only (13/39, 33%) or quick options only (5/39, 13%). There was a trend in which the proportion of CAs that supported free text along with quick options as user input increased, while the proportion of CAs that supported free text only as user input decreased over time (Figure 4).

##### Output Modality

All the CAs (39/39, 100%) supported text as the primary mode of output. In 64% (25/39) of the studies, it was indicated that CAs support multimedia outputs. In addition to textual output, most CAs (21/39, 54%) supported image output in the form of an emoji, moving images in graphics interchange format, infographics, and animated CA avatars. CAs also provided outputs in the forms of video (12/39, 31%), audio (7/39, 18%), and games (6/39, 15%). In many of the reviewed studies (12/39, 31%), CAs supported >2 types of output (eg, text, image, and video).

#### Many CAs Used Multiple AI Techniques: Free-Textual Inputs Were Processed via Natural Language Processing, While Prewritten Content Was Delivered via Rule-Based Programming

In many studies that we reviewed, the CAs used multiple AI-based computational methods depending on the specific section or feature. Free-textual input from users is processed using natural language processing (NLP), which processes natural language datasets, such as text and voice, using statistical and machine learning models to recognize, understand, and generate text and speech [58]. Accordingly, the AI approaches that were most frequently referred to in the reviewed papers were NLP (19/39, 49%). Meanwhile, quick options are implemented using rule-based methods. Therefore, following the NLP, the second most frequently referenced technique in the reviewed papers was rule-based approaches (15/39, 38%). Mostly, the CAs deliver prewritten conversational lessons via rule-based programming. However, they use techniques such as NLP at certain points in the tree to determine routing to subsequent conversational nodes (eg, [18,26,38,44,54,55]). For instance, NLP is used to process users’ input so that CAs can trigger prewritten therapeutic content [47] or further crises and mental health support services [26] to youth. There were a few papers in which CAs relied solely on decisions made by decision trees or rule-based programs (eg, [23,25,50,51]). With rule-based CAs, once users choose among the given options (eg, yes or no and choosing numbered options), the CAs trigger and provide the most relevant prewritten response. With the CAs that are built solely upon rule-based or decision tree techniques, there is no space for users to type free text to have conversations. None of the mental health CAs we reviewed were built upon generative AI such as large language models (LLMs).

#### Most Mental Health Content Was Delivered in Flexible Ways, Considering User Preferences and Autonomy

We found 3 types of CA content delivery approaches in the reviewed studies: structured, semistructured, and flexible. In most of the studies (25/39, 64%), mental health CAs implemented a flexible model. With a flexible model, CA contents are delivered depending on users’ input or users have options to choose which content to navigate. For instance, CAs first detect stress levels from users’ input; then, based on the stress detection result as well as the users’ chatting sentence type, the CA chooses an appropriate answer from the knowledge base database [44] or users have options to choose what type of CBT-based therapy they would like to perform [53].

In 20% (8/39) of the reviewed studies, CA content was delivered in a planned and structured way. With the structured models, CA contents are prewritten and automatically delivered to users via rule-based programming for a predefined period (ie, from a few days to a few weeks). For instance, Williams et al [38] designed a mental health CA in which daily conversations and modules are prewritten for 21 days, and the CA guides them through daily activity. Particularly, in half of the papers, the structured CA content was designed to treat mental health conditions of at-risk youth, including adolescent or young adults with depressive symptoms [29,51], youth treated for cancer [43], and adolescents with type 1 diabetes [26].

In a small portion of the reviewed studies (3/39, 8%), CA content was delivered in a semistructured way [32,48,49]. For instance, the mental health CA guided the user through 10 daily sessions that introduced the psychological content modules. The same core psychological concepts (eg, psychoeducation, emotional regulation, and problem-solving) are present for all users, while users can specify their preferences for the content that they feel is most relevant to them [49]. In another study, the mental health content spanning 21 days was structurally designed for users to engage with the CA once a day with a choice of a motivational quote, joke, or an entry into a gratitude journal [32].

#### Mental Health Content Was Built Upon Evidence-Based Expert Knowledge of Mental Health

In all studies, primary data sources were expert knowledge (39/39, 100%). In almost half of the reviewed studies (19/39, 49%) on mental health CAs, the CA content was developed based on CBT, one of the most extensively studied evidence-based psychotherapy for a wide range of mental health issues such as depression, stress, and general mental well-being [59]. For instance, building upon the principles of CBT, He et al [29] designed 7 modules of mental health CA (ie, cognitive distortions, self-esteem, mindfulness meditation, mental energy, natural connection, self-help, and loneliness) for youth participants to complete 1 module per day during the 1-week intervention period. Besides CBT, mental health CA content was based on positive psychology (7/39, 18%) and other therapeutic content (7/39, 18%), such as interpersonal psychotherapy [19,22], acceptance commitment therapy [23], perceptual control therapy [46,52], self-compassion program [26], and microintervention to improve body image [48]. Some mental health CA content was built upon inputs from school health service experts or counselors (6/39, 15%) and positive answers collected from the web forum [44]. In some studies (4/39, 10%), the source of mental health CA content was not specified.

In summary, we found that most mental health CAs were designed as older peers to provide therapeutic and or educational content to promote youth mental well-being. Most of the CAs were built upon multiple computational methods to flexibly deliver prewritten and evidence-based mental health content. Text was the primary communication mode, with the majority supporting multimedia output. Textbox 1 presents a summary of the key trends in the design and computational aspects of mental health CAs for youth.

**Key trends of the design and computational aspects of mental health conversational agents (CAs) for youth.**

**Aspects and key trends**

- Design
  - Target audience: designed for older and general youth populations
  - Health context and goals: designed to help alleviate mental health symptoms or to promote general mental well-being
  - CA role and characteristics: designed to be life coaches or older peer mentors with friendly and empathetic tones
  - Personalization: personalization of CA characters was not supported in most of the studies
  - Safety features: safety features were discussed in less than half of the studies

- Computational
  - Artificial intelligence technique: multiple computational methods (eg, natural language processing and rule based) depending on the specific features
  - Input modality: all CAs were text based, with a couple of them supporting audio input along with textual input. Most CAs supported free text along with quick options as user input
  - Output modality: all CAs supported text as the primary mode of output, with the majority supporting multimedia outputs
  - Content delivery: CA content was delivered in flexible ways, considering user preferences and autonomy. In some cases, prewritten therapeutic content was delivered in a structured manner
  - Data source: CA content was based on well-established evidence-based expert knowledge

### Evaluation Outcomes Reported in Research on Mental Health CAs for Youth

#### Most Studies Focused on Evaluating the Effectiveness and Acceptability of CAs

##### Effectiveness

In most of the evaluative research (17/39, 44%), the outcome variable was the effectiveness of mental health CAs (Table 4 and Multimedia Appendix 5 [4,18-55]). In those studies, prevalidated scales such as the PHQ and Generalized Anxiety Disorder Scale were used to measure the mental health conditions of the youth before and after the use of mental health CAs. For instance, to evaluate the effectiveness of mental health CA *Tess*, Klos et al [45] conducted a randomized controlled trial with 181 Argentinian college students. The PHQ-9 [60], a questionnaire comprising 9 items that evaluate the frequency and severity of depressive symptoms, and the Generalized Anxiety Disorder-7 [61], a 7-item scale that evaluates the frequency and severity of anxious thoughts and behaviors, were used to measure the changes in participants’ mental health [45].

**Table 4:** Evaluation outcomes of mental health CAs^a^ for youth (N=39).

Dimensions and codes			Value, n (%)		Key trends
**Dependent measures**					Most studies focused on evaluating the effectiveness and acceptability of CAs
	Effectiveness	17 (44)			
	Acceptability	17 (44)			
	Usability	14 (36)			
	User engagement	10 (26)			
	Validity or accuracy	2 (5)			
	Personalization	1 (3)			
**Strengths**					Easy access to useful mental health information communicated through friendly, empathetic, and humanlike responses was the major strength of mental health CAs
	Accessibility	14 (36)			
	Useful content	14 (36)			
	Interactivity or engaging	12 (31)			
	Anonymity or confidentiality	7 (18)			
	Empathetic or friendly responses	6 (15)			
	Easy to navigate	4 (10)			
	Humanlike interaction	2 (5)			
	Nonjudgmental	2 (5)			
**Weaknesses**					Limited or repetitive content, lack of human language understanding, and robotic responses were the critical weaknesses of mental health CAs
	Limited responses or content	13 (33)			
	Lack of natural language understanding	8 (21)			
	Lack of personalized content	7 (18)			
	Non-humanlike traits	7 (18)			
	Textual information with jargon	5 (13)			
	Inaccurate responses	5 (13)			
	Data privacy and confidentiality	5 (13)			
**Ethical considerations**					Ethical considerations such as privacy, confidentiality, and safety of CAs were addressed in a few studies
	Privacy and confidentiality	4 (10)			
	Safety	1 (3)			

^a^CA: conversational agent.

##### Acceptability

Another most evaluated outcome of mental health CAs was acceptability (17/39, 44%). Overall, current mental health CAs were perceived as beneficial and acceptable by not only youth but also health professionals [4]. For instance, semistructured interviews with 3 young adults with autism showed that they considered the mental health chatbot novel and they would be interested in using such systems [53]. In a study by Mariamo et al [24], yes or no questions were associated with a lower likelihood of response compared to multiple-response choice questions and a higher likelihood of response compared to open-ended questions.

##### Usability

Next, the usability of mental health CAs was evaluated in 36% (14/39) of the studies. For instance, Williams et al [38] engaged with 124 young adults in New Zealand to evaluate mental health CAs with 21-day stress detox modules and found that youth appreciated the interactivity, accessibility, and chatbot design, particularly due to the visualized content as it is engaging. Similarly, a week-long exploratory study with 20 rural-living LGBTQ+ youth confirmed that youth appreciated the colorful design with multimedia content of *REALbot* as well as the conversational flow to first ask users’ preferred names [50].

##### User Engagement

User engagement was also evaluated (10/39, 26%) through analyzing log data of human-CA interaction. For instance, Matheson et al [48] evaluated user engagement with the body image chatbot *Topity*, which was designed to deliver microinterventions for adolescents. The results from randomized controlled trials with 1715 Brazilian adolescents show that 79% of the participants completed the minimum intervention dose of 1 microintervention technique. In addition, most participants chose to receive guidance from a female avatar of Topity, compared to a male avatar [48].

##### Accuracy and Personalization

In a few studies, the accuracy or validity of mental health assessment (2/39, 5%) as well as personalization (1/39, 3%) were also explored. For instance, a month-long user test showed that the chatbot’s stress detection module achieved a precision rate of 78.34% and a recall rate of 76.12% [44]. In terms of personalization of mental health CA, while both experts and youth emphasized the need for autonomy to flexibly choose or change a module instead of a fixed schedule, experts emphasized the importance of planned therapeutic modules in a fixed sequence [19].

#### Easy Access to Useful Mental Health Information Communicated Through Friendly, Empathetic, and Humanlike Responses Were the Major Strengths of Mental Health CAs

In 67% (26/39) of the reviewed studies, the strengths of current mental health CAs for youth were addressed. The most frequently mentioned strengths of mental health CAs were 24/7 availability (14/39, 36%), followed by useful information and therapeutic content (14/39, 36%) and the ability to have interactive and engaging conversations with CAs (12/39, 31%). In some studies, participants pointed to the strengths of mental health CAs, such as perceived anonymity or confidentiality (7/39, 18%), empathetic and friendly responses generated by CAs (6/39, 15%), and easy to navigate (4/39, 10%). In fewer studies (4/39, 10%), participants liked nonjudgmental responses generated by CAs [23,43] delivered in multimedia output [27,54]. They also liked the sense of caring and support [34,40,41] provided by CAs. Some participants appreciated humanlike interaction with CAs[27,49], perceived CAs as smart and trustworthy friends [37], and sometimes formed connections or friendships with bots [38]. They also liked the availability of personalized support [4,29] delivered by personalized CA avatars [19].

#### Limited or Repetitive Content, Lack of Human Language Understanding, and Robotic Responses Were the Critical Weaknesses of Mental Health CAs

In 62% (24/39) of the reviewed studies, the weaknesses of the current mental health CAs were addressed. The most frequently addressed limitations in the reviewed studies are limited or repetitive content provided by CAs (13/39, 33%), lack of understanding of human input (8/39, 20%), and lack of personalized content (7/39, 18%). Some of the limitations of mental health CAs were related to non-humanlike traits (7/39, 18%), such as too fast responses generated by CAs [30,41], lack of empathy in their responses [37,39], lack of trust toward nonhuman agents [35], a feeling of loneliness or disconnect when interacting with a bot [38], and CAs being robotic [50,54] and not smart enough [50]. Some youth found CA content with too much textual information with jargon or inaccurate or unclear responses generated by CAs hard to understand (5/39, 13%), while some shared concerns for confidentiality and privacy of sensitive information (5/39, 13%, respectively). In some studies, participants expressed difficulties with the free-text input mode as they found it hard to express their feelings [52] and utterances [20] rather than speak naturally. In a few studies, technical limitations such as overall technical immaturity [29,41] and susceptibility to changes in platform policies and bugs [51,54] were addressed. Some of the potential safety concerns addressed in the reviewed studies were increased screen time [22], overreliance on machines over human support [4,22], risk of missing imminent risk [22,51], and age-appropriateness of CA content [25]. Table 5 shows a summary of the strengths and weaknesses of mental health CAs reported in the reviewed studies.

**Table 5:** Mapping of the strengths and weaknesses of mental health CAs^a^ for youth by dimension.

Dimensions	Strengths	Weaknesses
CA characteristic	Empathetic and friendly responses	Too fast responses without empathy
CA content	Useful information or therapeutic content	Inaccurate or unclear responses
AI^b^ technique	Interactive and engaging conversations	Limited or repetitive content and lack of understanding of human input
Input mode	Easy to start a conversation	Difficulties with terming the queries
Output mode	Multimedia output	Too much textual information with jargon
Personalization	Availability of personalized support	Lack of personalized content
Privacy and confidentiality	Perceived anonymity or confidentiality	Concerns for the privacy of sensitive information
Safety	Sense of caring and support via humanlike interaction with CAs	Potential for overreliance on CAs over human support and risk of missing imminent risk

^a^CA: conversational agent.

^b^AI: artificial intelligence.

#### Ethical Considerations Such as Privacy, Confidentiality, and Safety of Mental Health CAs Were Addressed in a Few Studies

Most of the reviewed studies (35/39, 90%) did not address the ethical aspects of mental health CAs. In 10% (4/39) of the included studies, ethical considerations such as privacy, confidentiality, and safety of CAs were addressed [4,22,30,35] as part of their empirical findings. For instance, safety was assessed at 2, 4, 8, and 12 weeks by parents’ reports on any hospitalizations or emergency department visits made by their child for depression- or anxiety-related problems. By the end of the 12-week experiment, 1 parent from the intervention group reported that their teen was seen in an emergency department and discharged to home [22], indicating the potential safety concerns and the need for features to ensure the safety of youth. Meanwhile, in 1 study, ethical issues (eg, privacy and confidentiality, efficacy, and safety of CAs) related to mental health CAs were discussed as the primary focus of the study. Through the group discussions with youth aged between 14 and 18 years in the United Kingdom, the authors highlighted youth’s concerns about mental health CAs related to their personal information. Their recommendations for designing ethical mental health CAs include (1) providing clear and transparent communication about the systems’ privacy arrangement and limitations, (2) informing users of the extent to which the chatbots are evidence based and empirically tested, and (3) ensuring that automated chatbots have systems in place to prevent overreliance and encourage users to seek human support when needed [39]. Overall, more discussion on ethics standards and critical reflections on mental health CAs for the youth is needed.

## Discussion

### Overview

In this scoping review, we identified 39 studies that focused on CAs designed to support the mental health of youth. In the subsequent sections, we discuss the implications of our findings and outline directions for future research. Finally, we provide recommendations for designing youth-centered, effective, and safe CA systems to support the mental health of youth.

### Principal Findings

#### Mental Health CAs That Can Support Diverse Youth With a Variety of Mental Health Issues Are Needed

In most of the reviewed studies, the target audience of mental health CAs was general youth, with a recent trend toward designing mental health CAs for at-risk youth populations. According to 2021 statistics, growing numbers of youth are at risk of poor mental health outcomes. For instance, nearly half (45%) of LGBTQ+ students seriously considered attempting suicide—far more than heterosexual students [62]. Accordingly, the US Surgeon General set an agenda to prioritize promoting the mental health of at-risk youth populations, such as racial, ethnic, sexual, and gender minority youth; individuals from lower socioeconomic backgrounds; youth with disabilities; youth involved in the juvenile justice system; and other groups [5]. Therefore, future work is needed to design and implement CAs to support the mental health of youth considered vulnerable. In addition, we found that most mental health CAs were designed to reduce symptoms of depression, anxiety, and stress, while only a few were designed to provide support for body image, phone addiction, substance use, and other mental health issues. A recent report shows that youth are increasingly experiencing diverse mental health issues, including attention-deficit/hyperactivity disorder, eating disorders, body image, suicide, and self-harm [5,62,63]. Therefore, mental health CAs that can support youth with a variety of mental health issues are needed.

#### Multimodal Input and Output Are Needed to Designing Mental Health CAs That Are Inclusive of Youth With Diverse Communication Needs

In terms of user input, all mental health CAs supported textual input, with very few supporting voice-based output. In most of the reviewed studies, mental health CAs supported free text along with quick options as user input, with a few supporting quick options only. Previous research showed that rule-based CAs with quick options are perceived as restricted in offering personalized advice, leading to low trust in the effectiveness of CAs in providing advice on sensitive topics [64]. Therefore, providing options to freely type queries could benefit youth to explore diverse mental health topics. At the same time, it would still be useful to have quick options to choose from or autofill features as some of the health topics are difficult to term the queries from scratch [65]. In addition, as some youth were frustrated by the need to type their utterances rather than speak naturally [20], an option for voice-based input methods could be beneficial for supporting youth with diverse communication needs. When it comes to output mode, in almost two-thirds of studies, mental health CAs provided content in the form of images, audio, video, or games along with textual information. Multimedia content provided by mental and health CAs is important, as evaluative research confirmed that some youth found lengthy texts hard to understand [37,54] and preferred interactive and engaging multimedia content [27]. Hence, along with textual information, providing health information in a multimedia format is needed for designing engaging CAs for youth.

#### More Advanced AI Technologies (eg, LLMs) Are Needed to Provide Interactive and Engaging Mental Health Support for Youth

Overall, mental health CAs for youth are in their infancy, as many of the systems are developed as prototypes and are being evaluated for improvement. One of the major limitations found in evaluative research was limited content or responses provided by CAs as well as a *lack of personalized content* (eg, [25,27,33,38]). This is because many of the mental health CAs were built upon a rule-based approach in which predefined sets of responses are based on domain-specific knowledge. Early evidence showed that rule-based CAs were seen as *only providing advice about mainstream*, easily accessible information that was already available on the internet [64]. The evaluative studies we reviewed also demonstrated that youth perceived mental health content provided by rule-based CAs to be *repetitive* (eg, [29,31,38,49]). However, many of the existing research studies implemented rule-based approaches to provide mental health information for youth. Another major technical limitation found in the evaluative research was the lack of human language understanding, followed by inaccurate responses from CAs. Taken together, our findings signify the need for implementing more sophisticated language models in mental health CA development. A recent review study found that CAs enhanced by advanced AI technologies outperformed rule-based CAs in managing psychological distress [11]. Further research is warranted to explore the potential benefits of implementing advanced AI technology (eg, generative AI) in mental health CAs for youth.

#### Safety Should Be Prioritized When Designing and Implementing Mental Health CAs for Youth

Recent advancements in LLMs are promising in improving the technical immaturity of mental health CAs, with the ability to understand input text written in human language in prompts and generate responses [66]. Early evidence demonstrated the effectiveness of the LLMs in generating coherent and relevant answers to psychological questions [67] or detecting mental health conditions [68]. However, when applying LLMs in mental health CAs for youth, the safety aspects of the information provided by those models should be rigorously considered, given the recent documentation of age-inappropriate and inaccurate content for youth generated by LLMs [69]. Therefore, chatbot development and implementations should undergo a robust validation process to establish a reliable and expert-informed evidence base for safety, particularly with extra care when designing chatbots for youth considered vulnerable (eg, those with mental health conditions). Meanwhile, safety concerns addressed in evaluative research included increased screen time, overreliance on machines over human support, and the risk of missing imminent risk. Although research findings are conflicting, the impact of overreliance on machines and increased screen time on youth’s well-being is one of the important safety concerns [70,71]. Therefore, safety features to help track screen time and nudge youth about their CA use could be considered. Safety features to alert mental health professionals for imminent risk (eg, nudges with sophisticated AI tech such as deep learning) could also be critical for mental health CAs.

#### Long-Term, Large-Scale, and Rigorous Evaluation Is Needed to Ensure the Efficacy and Safety of Mental Health CAs

Overall, most of the empirical research on mental health CAs was conducted with <100 older youth populations in the short term (<4 weeks). This trend was substantial as many of them involved user testing or clinical trials to assess the acceptability and feasibility of the prototypes. Although preliminary evidence shows positive trends in the effectiveness and acceptability of mental health CAs, long-term evaluative research with larger sample sizes and more robust research designs is needed to validate their efficacy before their widespread adoption and use. In addition, we noted a lack of established methods for evaluating the safety of mental health CAs for unintended adverse effects among reviewed studies. This concerning trend was consistent with the mental health CAs for general adults [72]. While empirical research showed that the age-appropriateness of mental health content was one of the weaknesses of mental health CAs [25], none of the reviewed studies evaluated whether the mental health content is developmentally appropriate for youth. Therefore, the accuracy and age-appropriateness of mental health content provided by CAs should be further explored to ensure the efficacy and safety of mental health CAs for youth.

#### Collaborative Efforts With Youth and Clinical Experts Are Needed to Design Safe, Effective, and Youth-Centered Mental Health CAs

The evaluation outcomes of the reviewed studies raised a few open questions in designing safe, effective, and youth-centered mental health CAs. One key issue is the humanlike traits of CAs; while youth prefer humanlike traits of CAs, existing research documented safety concerns toward the humanness of CAs. For instance, previous research shows that younger youth may lose vital human contact [73] or unintentionally share personal information with CAs [74] if they become too attached to humanlike CAs. Therefore, in some studies we reviewed, CAs were designed with non-humanlike avatars (eg, [23,25,30,34]) or there were safety features to clearly state that the CAs are not human agents (eg, [4,27,33,35,43,47,54]). Consequently, how to balance humanlike and non-humanlike traits of mental health CAs for youth is an important open question to address in collaborative research with youth and clinical experts.

Another open question is on the CA role, as evaluative research showed conflicting perceptions from youth and clinical experts; youth prefer peer- or coach-like roles, while experts were cautious about such roles for clinical purposes [19]. Therefore, careful consideration is needed when designing social roles of mental health CAs for youth taking into account specific health context, purpose, and target audience. In addition, as evaluative research showed, providing an option to choose a preferred CA avatar can help youth feel comfortable when sharing confidential issues on sensitive topics [37]. However, we found that the current empirical research on mental health CAs rarely explored how personalizing the roles and characteristics of mental health CAs impacts the effectiveness or user experience in both positive and negative ways. Therefore, further empirical research with youth and clinical experts is needed to understand how different permutations of CA roles and personalities play a role in supporting youth mental health.

In terms of personalized CA content delivery, we observed a trade-off between flexibility and structured planning in therapeutic content. Experts emphasized that planned modules could make mental health CAs more reliable and the treatment goals more visible; at the same time, they can reduce motivation and user engagement and thus lead to dropout [19]. Therefore, balancing between personalizing therapeutic content flexibly and maintaining a structured program is an open-ended question to address in future research. Finally, in most reviewed studies, the content of mental health CAs was based on well-established evidence-based expert knowledge to safely leverage CA systems. However, to understand the mental health needs of youth, it is equally critical to work with youth from the early stages of CA design. However, very little work has been done to consider inputs from youth in content generation. Taken together, more collaborative research efforts with youth, caregivers, and domain experts need to be made to address the aforementioned open questions and build effective, safe, and youth-centered mental health CAs.

#### Ethics Standards and Best Practices to Design and Develop Mental Health CAs for Youth Are Needed

We found a concerning trend in the existing literature on mental health CAs for youth: most of the papers did not address the ethical aspects of mental health CAs. Many of the existing review studies pointed to the lack of ethical considerations in the development of CAs in health care [2,75,76]. While personal health data are collected and processed in mental health CAs, the majority did not provide information regarding security and privacy aspects [76]. As mental health topics are sensitive, particularly for youth [6,30], more discussion on ethics standards and critical reflections on best practices to develop mental health CAs for the youth population is needed. Another concerning trend we found was that the ethical implications and best practices of involving youth considered vulnerable in evaluative research on mental health CAs are rarely discussed. In a 12-week evaluative research study with adolescents with depression and anxiety, more than half of the participants triggered at least 1 alarm for suicidal ideation [22], which signals that safety standards and best practices are pivotal when working with populations considered vulnerable. Hence, further research is needed to establish ethical standards for working with youth, particularly youth considered vulnerable, to ensure that participating in research does not harm already populations considered vulnerable. We summarize the open questions that need to be addressed in future research in Textbox 2.

**Open questions related to mental health conversational agents (CAs) to be addressed in future research.**

**Open questions**

- CA role: How do different CA roles and characteristics impact youth’s interaction with mental health CAs?
- CA characteristics: How can we balance humanlike versus non-humanlike traits of mental health CAs for youth?
- CA content: What is the age-appropriate, inclusive, and accurate mental health information for youth?
- Content delivery: What are the effective and safe delivery modes (ie, structured vs flexible) of mental health CA content for youth?
- Safety: How can we design CA features to promote the safety (eg, monitoring imminent risk and screen time) of mental health CAs?
- Data privacy and confidentiality: How can we design artificial intelligence–based systems that ensure privacy and confidentiality of youth data on sensitive topics?
- Research ethics: What are the ethics standards and best practices to design and develop mental health CAs for and with youth?

### Design Guidelines

On the basis of our findings and broader implications, we provide the following guidelines for designing youth-centered mental health CAs (Textbox 3).

**Design guidelines for mental health conversational agents (CAs) for youth.**

**Design guidelines**

- CA role and characteristics: The CA role should be carefully designed considering the purpose and primary target audience. Regardless of roles, empathetic and friendly characteristics of mental health CAs are important.
- CA content: CA content should be age-appropriate, inclusive, and accurate. The content should be based on evidence-based expert knowledge along with inputs from youth.
- Artificial intelligence technique: More advanced language models (eg, large language models) are needed to provide diverse, context-aware, and personalized content for youth.
- Input mode: Free-textual inputs with auto-complete or quick options can help youth formulate questions that require domain knowledge. Along with textual input, an option for voice-based input can support youth with diverse communication needs.
- Output mode: Less textual content and more multimedia content can help youth understand mental health information.
- Personalization: It is important to give youth control over the personalization of CA content and avatars. Information on what is being personalized and how it is done should be clear and transparent.
- Safety: It should be clearly communicated to youth that CAs are not human, along with information on the capability and limitations of CAs. Information on confidentiality and data privacy should be clear and transparent. Safety features such as emergency contacts for imminent risk should be provided upfront and available 24/7. Additional safety features to track screen time could help reduce overreliance on CAs and prevent excessive screen time.
- Ethics: Safety standards and critical reflections on best practices to co-design CAs with the youth population are needed.

### Strengths, Limitations, and Future Work

This scoping review has several strengths. First, we conducted a comprehensive literature search of multiple databases. We used holistic search terms rather than specific ones to capture the various representations of CAs used in mental health for youth. Second, we analyzed trends in empirical research on mental health CAs for youth as well as design and computational considerations of the mental health CAs studied in empirical research to provide a holistic mapping of the current landscape. Therefore, this study showcased the possible framework of mental health CAs that can be referenced by other researchers in this field.

However, our study has some limitations. First, given the novelty and multidisciplinary nature of the field, some unpublished literature presented at niche conferences and meetings may have been omitted. Second, during the data extraction process, we identified the design and computational approaches of the mental health CAs based on the descriptions reported in the reviewed studies. Hence, some of the design and computational aspects of mental health CAs that were considered yet not reported in the reviewed studies may not have been captured in this paper. As our findings suggest, the maturity of mental health CAs is still in its infancy, and further review with more in-depth analysis is needed as research in the field matures.

### Conclusions

CAs are increasingly used by youth for sensitive topics such as mental health. Trends in research on mental health CAs designed for youth have been underexplored. In this review paper, we fill an important gap by synthesizing 39 studies on mental health CAs designed for youth over the last 14 years. Our scoping review highlights that research on mental health CAs is in its infancy, and early evidence shows both strengths and weaknesses in existing systems. We call attention to important open questions that researchers should address to move forward. When designing CAs for youth, a one-size-fits-all approach does not apply. With careful consideration of the health context and needs of specific target groups, mental health CAs can benefit youth. This can be only achieved when engaging with youth from the early design phases to the summative evaluation of the systems. In this regard, we call for further investigation of best practices for risk mitigation strategies and ethical development of CAs with and for youth to promote their mental well-being.

## Appendix

Multimedia Appendix 1 PRISMA-ScR (Preferred Reporting Items for Systematic Reviews and Meta-Analyses Extension for Scoping Reviews) checklist.

Multimedia Appendix 2 Characteristics of the included studies.

Multimedia Appendix 3 Design considerations for mental health conversational agents for youth.

Multimedia Appendix 4 Computational considerations of mental health conversational agents for youth.

Multimedia Appendix 5 Evaluation outcomes of the included studies.

## Abbreviations

AI: artificial intelligence
CA: conversational agent
CBT: cognitive behavioral therapy
LGBTQ+: lesbian, gay, bisexual, transgender, queer, or questioning
LLM: large language model
NLP: natural language processing
PHQ: Patient Health Questionnaire
PRISMA: Preferred Reporting Items for Systematic Reviews and Meta-Analyses
RQ: research question
