# Supplementary material for: Current Landscape and Future Directions for Mental Health Conversational Agents for Youth: Scoping Review
Source: JMIR Med Inform. 2025 Feb 28;13:e62758. doi: 10.2196/62758 (PMC11909484; doi:10.2196/62758)
Supplement: Multimedia Appendix 3 [file medinform_v13i1e62758_app3.docx]

**Multimedia Appendix 3. Design considerations of mental health conversational agents for youth.**

| **References** | **Target Audience** | **Health Context** | **CA Goals** | **CA Role** | **CA Characteristics** | **Personalization** | **Safety Features** |
| --- | --- | --- | --- | --- | --- | --- | --- |
| Koulouri et al. [1] | Young adults | Mental well-being | Assessment, Treatment, Education/Training, Information | Health professional | Empathetic | Did not specify | Reminder that they do not replace a therapist |
| Fitzpatrick et al. [2] | Young adults with symptoms of depression and anxiety | Depression, Anxiety | Treatment | Health professional | Empathetic | Did not specify | Reminder that the service should not be used as a replacement for therapy, Encourage users to call 911 for emergencies |
| Kuhlmeier et al. [3] | Youth and Young adults with depression | Depression | Treatment | Coachlike | Friendly | CA character, CA content | Did not specify |
| Elmasri et al. [4] | Young adults | Alcohol abuse, Assessment | Information, Education/Training, Assessment, Behavioral Change | Coachlike | Friendly | Did not specify | Did not specify |
| Abreu et al. [5] | Young adults | Phone addiction | Behavior Change | Did not specify | Friendly | Did not specify | Did not specify |
| Nicol et al. [6] | Adolescents | Depression | Treatment, Education/Training | Did not specify | Did not specify | Did not specify | Reminder that the app is not a crisis intervention, Crisis hotline number, Safety measures to alert providers |
| Beilharz et al. [7] | Young people with concerns with body image and eating issues | Body image | Education, Treatment, Information | Health professional | Short and simple, | Did not specify | Reminder that the app is not a crisis intervention, Helpline, crisis support services |
| Mariamo et al. [8] | Adolescents | Mental well-being | Did not specify | Did not specify | Did not specify | Did not specify | Did not specify |
| Dosovitsky et al. [9] | Adolescents | Depression | Treatment, Education/Training | Did not specify | Did not specify | Did not specify | Did not specify |
| Boggiss et al. [10] | T1D adolescents | Mental well-being | Education/Training, Treatment, Information | Did not specify | Did not specify | CA character | Did not specify |
| Gabrielli et al. [11] | Adolescents | Mental well-being | Education/Training | Older peer | Did not specify | CA character | Did not specify |
| Schick et al. [12] | Adolescents and young adults | Stress, Alcohol abuse | Assessment | Did not specify | Did not specify | Did not specify | Did not specify |
| He et al. [13] | Young adults with depressive symptoms | Depression | Assessment, Education, Treatment, Information | Did not specify | Did not specify | Did not specify | Did not specify |
| Sanabria et al. [14] | Youth at risk of HIV and STIs | Mental well-being | Assessment, Information | Did not specify | Friendly | Did not specify | Reminder that the app is not a crisis intervention, Crisis hotline number |
| Grové et al. [15] | Young people | Mental wellbeing | Education/Training, Information | Did not specify | Did not specify | Did not specify | Alert feature to notify a trusting adult when youth was identified as risk to themselves or others |
| Holt-Quick et al. [16] | Youth | Mental well-being | Treatment, Education/Training | Coachlike (older peer) | Culture specific | CA character | Did not specify |
| Ludin et al. [17] | Young people | Mental wellbeing | Assessment, Treatment, Education/training | Older peer | Culture specific, Gender specific | Did not specify | Reminder that the Aroha character is not a real person |
| Høiland et al. [18] | Youth | Mental well-being | Education, information | Coachlike (peer-like) | Friendly, Empathetic | Did not specify | Did not specify |
| Brandtzæg et al. [19] | Young people | Mental well-being | Treatment, Education/Training, Information | Did not specify | Friendly, Empathetic | Did not specify | Reminder that the chatbot is not a real person and don't use this as a substitute for getting help |
| Oliveira et al. [20] | College students | Depression, Anxiety | Treatment, Education/Training | Did not specify | Did not specify | Did not specify | Providing users with emergency contacts and encouraging users to call emergency numbers in case of emergencies. |
| Gabrielli et al. [21] | University students | Anxiety, Stress | Treatment, Education/Training | Coachlike | Empathetic | Did not specify | Reminder that the chatbot is not a replacement of professional mental health treatment |
| Williams et al. [22] | Young adults | Anxiety, Stress | Treatment, Education/Training | Peer | Friendly, Empathetic, Simple | Did not specify | Risk keywords programmed triggers a "more help" module to present helplines and support. |
| Kretzschmar et al. [23] | Young people | Mental health | Treatment | Did not specify | Did not specify | Did not specify | Did not specify |
| De Nieva et al. [24] | Senior High | Stress | Treatment, Education/Training | Health professional | Friendly, Empathetic | Did not specify | Did not specify |
| Maenhout et al. [25] | Adolescents | Mental well-being | Information | Health professional | Friendly, Empathetic | Did not specify | Referrals to professional help when needed |
| Crutzen et al. [26] | Adolescents | Sex, Drug, Alcohol | Information | Did not specify | Did not specify | Did not specify | Did not specify |
| Greer et al. [27] | Young people treated for cancer | Depression, Anxiety | Treatment, Education/Training | Did not specify | Did not specify | Did not specify | Reminder that the chatbot is not a real person |
| Huang et al. [28] | Adolescents | Stress, | Assessment, Treatment | Did not specify | Did not specify | Did not specify | Did not specify |
| Klos et al. [29] | University students | Depression, Anxiety | Treatment | Did not specify | Friendly, Empathetic, Culture specific | Did not specify | Provide National Line of Suicide Prevention numbers for participant with suicidal ideation, crisis text line, and 911 and encourage seeking professional help |
| Gaffney et al. [30] | College students | Depression, Anxiety, Stress | Treatment | Healthcare professional | Did not specify | Did not specify | Did not specify |
| Liu et al. [31] | University students | Depression | Treatment, Assessment | Did not specify | Friendly, Empathetic | Did not specify | Reminder that the chatbot is not a real person |
| Matheson et al. [32] | Adolescents | Body image | Treatment, Education/Training | Did not specify | Friendly, Gender specific | CA gender | Did not specify |
| Fabian et al. [33] | Young adults from immigrant, refugee communities | Transdiagnostic (depression, anxiety, etc.) | Treatment, Education/Training | Did not specify | Friendly | Did not specify | Did not specify |
| Escobar-Viera et al. [34] | LGBTG+ youth | Mental well-being | Education/Training, Monitoring, Information | Did not specify | Friendly | Did not specify | Did not specify |
| Viduani et al. [35] | Adolescents with depression | Depression | Assessment, Monitoring | Did not specify | Friendly | Did not specify | Reminder that chatbot was not a channel for seeking help, Mental health support from researchers, Helplines and emergency contacts. |
| Wrightson-Hester et al. [36] | Young people experiencing symptoms of anxiety, depression, or low mood | Depression, Anxiety, Stress | Treatment, Information | Healthcare professional | Did not specify | User avatar | Did not specify |
| Palma et al. [37] | Young adults with Autism | Anxiety, Stress | Treatment | Did not specify | Empathetic, Friendly | CA avatar, App appearance | Did not specify |
| Kang et al. [38] | Young people in Aotearoa | Mental wellbeing | Information | Older peer | Culture specific, Gender specific | Did not specify | Reminder that the Aroha character is not a real person |
| Afrin et al. [39] | Adolescents | Mental well-being | Assessment, Information, Education | Did not specify | Did not specify | Did not specify | Providing users with human experts and emergency contacts |

References

[1] Koulouri T, Macredie RD, Olakitan D. Chatbots to support young adults’ mental health: An exploratory study of acceptability. ACM Transactions on Interactive Intelligent Systems (TiiS). 2022;12(2):1-39. doi: 10.1145/3485874

[2] Fitzpatrick KK, Darcy A, Vierhile M. Delivering Cognitive Behavior Therapy to Young Adults With Symptoms of Depression and Anxiety Using a Fully Automated Conversational Agent (Woebot): A Randomized Controlled Trial. JMIR Ment Health. 2017 Jun 06;4(2):e19. PMID: 28588005. doi: 10.2196/mental.7785.

[3] Kuhlmeier FO, Gnewuch U, Lüttke S, Brakemeier E-L, Mädche A. A Personalized Conversational Agent to Treat Depression in Youth and Young Adults – A Transdisciplinary Design Science Research Project. Lecture Notes in Computer Science (including subseries Lecture Notes in Artificial Intelligence and Lecture Notes in Bioinformatics); 2022. doi: 10.1007/978-3-031-06516-3_3

[4] Elmasri D, Maeder A, editors. A conversational agent for an online mental health intervention. Brain Informatics and Health: International Conference, BIH 2016, Omaha, NE, USA, October 13-16, 2016 Proceedings; 2016: Springer. doi: 10.1007/978-3-319-47103-7_24

[5] Abreu C, Campos PF, editors. Raising awareness of smartphone overuse among university students: a persuasive systems approach. Informatics; 2022: MDPI. doi: 10.3390/informatics9010015

[6] Nicol G, Wang R, Graham S, Dodd S, Garbutt J. Chatbot-Delivered Cognitive Behavioral Therapy in Adolescents With Depression and Anxiety During the COVID-19 Pandemic: Feasibility and Acceptability Study. JMIR Form Res. 2022 Nov 22;6(11):e40242. PMID: 36413390. doi: 10.2196/40242.

[7] Beilharz F, Sukunesan S, Rossell SL, Kulkarni J, Sharp G. Development of a Positive Body Image Chatbot (KIT) With Young People and Parents/Carers: Qualitative Focus Group Study. J Med Internet Res. 2021 Jun 16;23(6):e27807. PMID: 34132644. doi: 10.2196/27807.

[8] Mariamo A, Temcheff CE, Léger PM, Senecal S, Lau MA. Emotional Reactions and Likelihood of Response to Questions Designed for a Mental Health Chatbot Among Adolescents: Experimental Study. JMIR Hum Factors. 2021 Mar 18;8(1):e24343. PMID: 33734089. doi: 10.2196/24343.

[9] Dosovitsky G, Bunge E. Development of a chatbot for depression: adolescent perceptions and recommendations. Child Adolesc Ment Health. 2023 Feb;28(1):124-7. PMID: 36507594. doi: 10.1111/camh.12627.

[10] Boggiss A, Consedine N, Hopkins S, Silvester C, Jefferies C, Hofman P, et al. Improving the Well-being of Adolescents With Type 1 Diabetes During the COVID-19 Pandemic: Qualitative Study Exploring Acceptability and Clinical Usability of a Self-compassion Chatbot. JMIR Diabetes. 2023 May 05;8:e40641. PMID: 36939680. doi: 10.2196/40641.

[11] Gabrielli S, Rizzi S, Bassi G, Carbone S, Maimone R, Marchesoni M, et al. Engagement and Effectiveness of a Healthy-Coping Intervention via Chatbot for University Students During the COVID-19 Pandemic: Mixed Methods Proof-of-Concept Study. JMIR Mhealth Uhealth. 2021 May 28;9(5):e27965. PMID: 33950849. doi: 10.2196/27965.

[12] Schick A, Feine J, Morana S, Maedche A, Reininghaus U. Validity of Chatbot Use for Mental Health Assessment: Experimental Study. JMIR Mhealth Uhealth. 2022 Oct 31;10(10):e28082. PMID: 36315228. doi: 10.2196/28082.

[13] He Y, Yang L, Zhu X, Wu B, Zhang S, Qian C, et al. Mental Health Chatbot for Young Adults With Depressive Symptoms During the COVID-19 Pandemic: Single-Blind, Three-Arm Randomized Controlled Trial. J Med Internet Res. 2022 Nov 21;24(11):e40719. PMID: 36355633. doi: 10.2196/40719.

[14] Sanabria G, Greene KY, Tran JT, Gilyard S, DiGiovanni L, Emmanuel PJ, et al. "A Great Way to Start the Conversation": Evidence for the Use of an Adolescent Mental Health Chatbot Navigator for Youth at Risk of HIV and Other STIs. J Technol Behav Sci. 2023 May 11:1-10. PMID: 37362063. doi: 10.1007/s41347-023-00315-4.

[15] Grové C. Co-developing a Mental Health and Wellbeing Chatbot With and for Young People. Front Psychiatry. 2020;11:606041. PMID: 33597898. doi: 10.3389/fpsyt.2020.606041.

[16] Holt-Quick C, Warren J, Stasiak K, Williams R, Christie G, Hetrick S, et al. A Chatbot Architecture for Promoting Youth Resilience. Healthier Lives, Digitally Enabled: IOS Press; 2021. p. 99-105. doi: 10.3233/SHTI210017

[17] Ludin N, Holt-Quick C, Hopkins S, Stasiak K, Hetrick S, Warren J, et al. A Chatbot to Support Young People During the COVID-19 Pandemic in New Zealand: Evaluation of the Real-World Rollout of an Open Trial. J Med Internet Res. 2022 Nov 04;24(11):e38743. PMID: 36219754. doi: 10.2196/38743.

[18] Høiland CG, Følstad A, Karahasanovic A. Hi, can I help? Exploring how to design a mental health chatbot for youths. Human Technology. 2020;16(2):139-69. doi:10.17011/ht/urn.202008245640

[19] Brandtzæg PB, Skjuve M, Kristoffer Dysthe KK, Følstad A, editors. When the social becomes non-human: young people's perception of social support in chatbots. Proceedings of the 2021 CHI conference on human factors in computing systems; 2021. doi: 10.1145/3411764.3445318

[20] Oliveira ALS, Matos LN, Junior MC, Delabrida ZNC, editors. An Initial Assessment of a Chatbot for Rumination-Focused Cognitive Behavioral Therapy (RFCBT) in College Students. Computational Science and Its Applications–ICCSA 2021: 21st International Conference, Cagliari, Italy, September 13–16, 2021, Proceedings, Part VI 21; 2021: Springer. doi: 10.1007/978-3-030-86979-3_39

[21] Gabrielli S, Rizzi S, Carbone S, Donisi V. A Chatbot-Based Coaching Intervention for Adolescents to Promote Life Skills: Pilot Study. JMIR Hum Factors. 2020 Feb 14;7(1):e16762. PMID: 32130128. doi: 10.2196/16762.

[22] Williams R, Hopkins S, Frampton C, Holt-Quick C, Merry SN, Stasiak K. 21-day stress detox: open trial of a universal well-being chatbot for young adults. Social Sciences. 2021;10(11):416. doi: 10.3390/socsci10110416

[23] Kretzschmar K, Tyroll H, Pavarini G, Manzini A, Singh I, Group NYPsA. Can Your Phone Be Your Therapist? Young People's Ethical Perspectives on the Use of Fully Automated Conversational Agents (Chatbots) in Mental Health Support. Biomed Inform Insights. 2019;11:1178222619829083. PMID: 30858710. doi: 10.1177/1178222619829083.

[24] De Nieva JO, Joaquin JA, Tan CB, Marc Te RK, Ong E, editors. Investigating students’ use of a mental health chatbot to alleviate academic stress. 6th International ACM In-Cooperation HCI and UX Conference; 2020. doi: 10.1145/3431656.3431657

[25] Maenhout L, Peuters C, Cardon G, Compernolle S, Crombez G, DeSmet A. Participatory Development and Pilot Testing of an Adolescent Health Promotion Chatbot. Front Public Health. 2021;9:724779. PMID: 34858919. doi: 10.3389/fpubh.2021.724779.

[26] Crutzen R, Peters GJ, Portugal SD, Fisser EM, Grolleman JJ. An artificially intelligent chat agent that answers adolescents' questions related to sex, drugs, and alcohol: an exploratory study. J Adolesc Health. 2011 May;48(5):514-9. PMID: 21501812. doi: 10.1016/j.jadohealth.2010.09.002.

[27] Greer S, Ramo D, Chang YJ, Fu M, Moskowitz J, Haritatos J. Use of the Chatbot "Vivibot" to Deliver Positive Psychology Skills and Promote Well-Being Among Young People After Cancer Treatment: Randomized Controlled Feasibility Trial. JMIR Mhealth Uhealth. 2019 Oct 31;7(10):e15018. PMID: 31674920. doi: 10.2196/15018.

[28] Huang J, Li Q, Xue Y, Cheng T, Xu S, Jia J, et al., editors. Teenchat: a chatterbot system for sensing and releasing adolescents’ stress. Health Information Science: 4th International Conference, HIS 2015, Melbourne, Australia, May 28-30, 2015, Proceedings 4; 2015: Springer. doi: 10.1007/978-3-319-19156-0_14

[29] Klos MC, Escoredo M, Joerin A, Lemos VN, Rauws M, Bunge EL. Artificial Intelligence-Based Chatbot for Anxiety and Depression in University Students: Pilot Randomized Controlled Trial. JMIR Form Res. 2021 Aug 12;5(8):e20678. PMID: 34092548. doi: 10.2196/20678.

[30] Gaffney H, Mansell W, Edwards R, Wright J. Manage Your Life Online (MYLO): a pilot trial of a conversational computer-based intervention for problem solving in a student sample. Behav Cogn Psychother. 2014 Nov;42(6):731-46. PMID: 23899405. doi: 10.1017/S135246581300060X.

[31] Liu H, Peng H, Song X, Xu C, Zhang M. Using AI chatbots to provide self-help depression interventions for university students: A randomized trial of effectiveness. Internet Interv. 2022 Mar;27:100495. PMID: 35059305. doi: 10.1016/j.invent.2022.100495.

[32] Matheson EL, Smith HG, Amaral ACS, Meireles JFF, Almeida MC, Linardon J, et al. Using Chatbot Technology to Improve Brazilian Adolescents' Body Image and Mental Health at Scale: Randomized Controlled Trial. JMIR Mhealth Uhealth. 2023 Jun 19;11:e39934. PMID: 37335604. doi: 10.2196/39934.

[33] Fabian KE, Foster KT, Chwastiak L, Turner M, Wagenaar BH. Adapting a transdiagnostic digital mental health intervention for use among immigrant and refugee youth in Seattle: a human-centered design approach. Transl Behav Med. 2023 Nov 05;13(11):867-75. PMID: 37418614. doi: 10.1093/tbm/ibad041.

[34] Escobar-Viera CG, Porta G, Coulter RWS, Martina J, Goldbach J, Rollman BL. A chatbot-delivered intervention for optimizing social media use and reducing perceived isolation among rural-living LGBTQ+ youth: Development, acceptability, usability, satisfaction, and utility. Internet Interv. 2023 Dec;34:100668. PMID: 37746640. doi: 10.1016/j.invent.2023.100668.

[35] Viduani A, Cosenza V, Fisher HL, Buchweitz C, Piccin J, Pereira R, et al. Assessing Mood With the Identifying Depression Early in Adolescence Chatbot (IDEABot): Development and Implementation Study. JMIR Hum Factors. 2023 Aug 07;10:e44388. PMID: 37548996. doi: 10.2196/44388.

[36] Wrightson-Hester AR, Anderson G, Dunstan J, McEvoy PM, Sutton CJ, Myers B, et al. An Artificial Therapist (Manage Your Life Online) to Support the Mental Health of Youth: Co-Design and Case Series. JMIR Hum Factors. 2023 Jul 21;10:e46849. PMID: 37477969. doi: 10.2196/46849.

[37] Palma R, Lam HC, Shrivastava A, Karlinsey E, Nguyen K, Deol P, et al., editors. “Monday Feels Like Friday!”-Towards Overcoming Anxiety and Stress of Autistic Young Adults During Times of Isolation. International Conference on Information; 2023: Springer. doi: 10.1007/978-3-031-28032-0_24

[38] Kang A, Hetrick S, Cargo T, Hopkins S, Ludin N, Bodmer S, et al. Exploring Young Adults' Views About Aroha, a Chatbot for Stress Associated With the COVID-19 Pandemic: Interview Study Among Students. JMIR Form Res. 2023 Oct 12;7:e44556. PMID: 37527545. doi: 10.2196/44556.

[39] Afrin Z, Farid DM, Mamun KAA, editors. A Cloud-Based Intelligent Virtual Assistant for Adolescents. International Conference on Intelligent Systems and Data Science; 2023: Springer. doi: 10.1007/978-981-99-7649-2_9
