# Supplementary material for: Current Landscape and Future Directions for Mental Health Conversational Agents for Youth: Scoping Review
Source: JMIR Med Inform. 2025 Feb 28;13:e62758. doi: 10.2196/62758 (PMC11909484; doi:10.2196/62758)
Supplement: Multimedia Appendix 4 [file medinform_v13i1e62758_app4.docx]

**Multimedia Appendix 4. Computational considerations of mental health conversational agents for youth.**

| **References** | **Maturity of Device** | **Delivery Channel** | **Input Mode** | **Output Mode** | **AI-Tech** | **Content Delivery** | **Knowledge Base** |
| --- | --- | --- | --- | --- | --- | --- | --- |
| Koulouri et al. [1] | Prototype | Facebook messenger (web, mobile) | Free text, Options | Text, Audio, Video | NLP | Flexible | CBT |
| Fitzpatrick et al. [2] | Existing system | Desktop, Mobile | Free text, Options | Text, Image, Video, Game | NLP, Rule-based | Flexible | CBT |
| Kuhlmeier et al. [3] | Prototype | Mobile | Free text, Options | Text, Image | Did not specify | Flexible | CBT, IPT |
| Elmasri et al. [4] | Prototype | Did not specify | Free text | Did not specify | NLP | Flexible | AUDIT-C |
| Abreu et al. [5] | Fully functioning system | Mobile | Free text | Did not specify | NLP | Flexible | Did not specify |
| Nicol et al. [6] | Existing system | Mobile | Free text | Did not specify | NLP, ML | Flexible | CBT, IPT-A, DBT |
| Beilharz et al. [7] | Prototype | Desktop, Mobile, Web | Options | Text, Image | Rule-based | Flexible | CBT, ACT, mindfulness |
| Mariamo et al. [8] | Design concept | Did not specify | Did not specify | Text, Image | Did not specify | Did not specify | Did not specify |
| Dosovitsky et al. [9] | Prototype | Facebook messenger (web) | Options | Did not specify | Rule-based | Structured | CBT |
| Boggiss et al. [10] | Prototype | Did not specify | Free text, Options | Text, Image, Audio, Video, Games | Rule-based, NLP | Structured | Self-compassion program |
| Gabrielli et al. [11] | Prototype, Fully functioning system | Desktop, Mobile, Tablet | Free text, Options | Text, Video | Did not specify | Flexible | Did not specify |
| Schick et al. [12] | Prototype | Desktop | Free text, Options | Did not specify | NLP | Did not specify | Did not specify |
| He et al. [13] | Fully functioning system | Web, Mobile | Did not specify | Text, Image, Audio | NLP, Deep Learning | Structured | CBT |
| Sanabria et al. [14] | Prototype | Desktop | Free text, Options | Text, Image | Did not specify | Flexible | Did not specify |
| Grové et al. [15] | Prototype | Did not specify | Free text | Did not specify | NLP | Flexible | Evidence-based strategies collected from clinical experts |
| Holt-Quick et al. [16] | Prototype | Facebook messenger (mobile) | Free text, Options | Text, Image | Rule-based | Semi-structured | CBT, Positive psychology |
| Ludin et al. [17] | Prototype | Facebook messenger (web, mobile) | Free text, Options | Text, Image, Audio, Video, Game | NLP | Flexible | CBT, Positive psychology, COVID-specific content (school counsellors, clinicians) |
| Høiland et al. [18] | Prototype | Mobile | Free text, Options | Text, Image | Did not specify | Flexible | Content development with school health service experts |
| Brandtzæg et al. [19] | Existing system | Did not specify | Free text, Options | Text, Image Video, Game | Did not specify | Flexible | CBT |
| Oliveira et al. [20] | Prototype | Facebook messenger (web, mobile) | Free text | Did not specify | NLP | Structured | Rumination-focused CBT (RFCBT), Dialogue from psychologist |
| Gabrielli et al. [21] | Prototype | Telegram app (mobile) | Options | Text, Video | Did not specify | Structured | CBT, Positive psychology by clinical psychologists |
| Williams et al. [22] | Prototype | Facebook messenger (mobile, desktop) | Free text, Options | Text, Image, Audio, Game | Rule-based, NLP | Structured | CBT, Positive psychology |
| Kretzschmar et al. [23] | Existing system | Did not specify | Free text | Did not specify | Did not specify | Did not specify | CBT |
| De Nieva et al. [24] | Existing system | Mobile | Free text | Text, Video, Image | Did not specify | Flexible | CBT |
| Maenhout et al. [25] | Prototype | Mobile | Free text | Did not specify | Did not specify | Flexible | Evidence-based (prior literature) |
| Crutzen et al. [26] | Existing system | Windows Live Messenger (web) | Free text | Did not specify | Did not specify | Flexible | National health promotion institutions in the Netherlands |
| Greer et al. [27] | Prototype | Facebook messenger | Free text, Options | Text, Video | Rule-based | Structured | Positive psychology |
| Huang et al. [28] | Prototype | Did not specify | Free text | Did not specify | NLP, Rule-based | Flexible | Positive answers from online forums and micro-blog/tweet |
| Klos et al. [29] | Existing system | Facebook messenger | Free text | Text, Image | Did not specify | Flexible | CBT |
| Gaffney et al. [30] | Prototype | Web | Free text | Did not specify | NLP | Flexible | PCT-based (Methods of Levels) |
| Liu et al. [31] | Prototype | WeChat (mobile) | Free text, Options, Speech | Did not specify | NLP, Rule-based | Flexible | CBT |
| Matheson et al. [32] | Fully functioning system | Facebook messenger | Free text, Options | Text, Image, Video, Games | Rule-based | Semi-Structured | Microintervention techniques, Body-image interventions |
| Fabian et al. [33] | Prototype | Facebook Messenger, WhatsApp | Free text, Options | Text, Image | Rule-based | Semi-Structured | Psychotherapy |
| Escobar-Viera et al. [34] | Fully functioning system | Facebook messenger (mobile, web) | Options | Text, Video, Image | Rule-based | Flexible | Evidence-based (prior literature) |
| Viduani et al. [35] | Prototype, Fully functioning system | WhatsApp | Options, Speech | Text, Image | Rule-based | Structured | Evidence-based (prior literature) |
| Wrightson-Hester et al. [36] | Prototype | Web, Mobile | Free text, Options | Did not specify | NLP | Flexible | PCT-based (Methods of Levels) |
| Palma et al. [37] | Prototype | Did not specify | Free text | Text, Image, Audio | NLP | Flexible | CBT-based |
| Kang et al. [38] | Fully functioning system | Facebook messenger (web, mobile) | Free text, Options | Text, Image, Audio, Video, Game | Rule-based, NLP | Flexible | CBT, Positive psychology, COVID-specific content (school counsellors, clinicians) |
| Afrin et al. [39] | Prototype | Mobile | Free text, Options | Text, Image | NLP, Rule-based, ML | Flexible | Evidence-based (prior literature) |

References

[1] Koulouri T, Macredie RD, Olakitan D. Chatbots to support young adults’ mental health: An exploratory study of acceptability. ACM Transactions on Interactive Intelligent Systems (TiiS). 2022;12(2):1-39. doi: 10.1145/3485874

[2] Fitzpatrick KK, Darcy A, Vierhile M. Delivering Cognitive Behavior Therapy to Young Adults With Symptoms of Depression and Anxiety Using a Fully Automated Conversational Agent (Woebot): A Randomized Controlled Trial. JMIR Ment Health. 2017 Jun 06;4(2):e19. PMID: 28588005. doi: 10.2196/mental.7785.

[3] Kuhlmeier FO, Gnewuch U, Lüttke S, Brakemeier E-L, Mädche A. A Personalized Conversational Agent to Treat Depression in Youth and Young Adults – A Transdisciplinary Design Science Research Project. Lecture Notes in Computer Science (including subseries Lecture Notes in Artificial Intelligence and Lecture Notes in Bioinformatics); 2022. doi: 10.1007/978-3-031-06516-3_3

[4] Elmasri D, Maeder A, editors. A conversational agent for an online mental health intervention. Brain Informatics and Health: International Conference, BIH 2016, Omaha, NE, USA, October 13-16, 2016 Proceedings; 2016: Springer. doi: 10.1007/978-3-319-47103-7_24

[5] Abreu C, Campos PF, editors. Raising awareness of smartphone overuse among university students: a persuasive systems approach. Informatics; 2022: MDPI. doi: 10.3390/informatics9010015

[6] Nicol G, Wang R, Graham S, Dodd S, Garbutt J. Chatbot-Delivered Cognitive Behavioral Therapy in Adolescents With Depression and Anxiety During the COVID-19 Pandemic: Feasibility and Acceptability Study. JMIR Form Res. 2022 Nov 22;6(11):e40242. PMID: 36413390. doi: 10.2196/40242.

[7] Beilharz F, Sukunesan S, Rossell SL, Kulkarni J, Sharp G. Development of a Positive Body Image Chatbot (KIT) With Young People and Parents/Carers: Qualitative Focus Group Study. J Med Internet Res. 2021 Jun 16;23(6):e27807. PMID: 34132644. doi: 10.2196/27807.

[8] Mariamo A, Temcheff CE, Léger PM, Senecal S, Lau MA. Emotional Reactions and Likelihood of Response to Questions Designed for a Mental Health Chatbot Among Adolescents: Experimental Study. JMIR Hum Factors. 2021 Mar 18;8(1):e24343. PMID: 33734089. doi: 10.2196/24343.

[9] Dosovitsky G, Bunge E. Development of a chatbot for depression: adolescent perceptions and recommendations. Child Adolesc Ment Health. 2023 Feb;28(1):124-7. PMID: 36507594. doi: 10.1111/camh.12627.

[10] Boggiss A, Consedine N, Hopkins S, Silvester C, Jefferies C, Hofman P, et al. Improving the Well-being of Adolescents With Type 1 Diabetes During the COVID-19 Pandemic: Qualitative Study Exploring Acceptability and Clinical Usability of a Self-compassion Chatbot. JMIR Diabetes. 2023 May 05;8:e40641. PMID: 36939680. doi: 10.2196/40641.

[11] Gabrielli S, Rizzi S, Bassi G, Carbone S, Maimone R, Marchesoni M, et al. Engagement and Effectiveness of a Healthy-Coping Intervention via Chatbot for University Students During the COVID-19 Pandemic: Mixed Methods Proof-of-Concept Study. JMIR Mhealth Uhealth. 2021 May 28;9(5):e27965. PMID: 33950849. doi: 10.2196/27965.

[12] Schick A, Feine J, Morana S, Maedche A, Reininghaus U. Validity of Chatbot Use for Mental Health Assessment: Experimental Study. JMIR Mhealth Uhealth. 2022 Oct 31;10(10):e28082. PMID: 36315228. doi: 10.2196/28082.

[13] He Y, Yang L, Zhu X, Wu B, Zhang S, Qian C, et al. Mental Health Chatbot for Young Adults With Depressive Symptoms During the COVID-19 Pandemic: Single-Blind, Three-Arm Randomized Controlled Trial. J Med Internet Res. 2022 Nov 21;24(11):e40719. PMID: 36355633. doi: 10.2196/40719.

[14] Sanabria G, Greene KY, Tran JT, Gilyard S, DiGiovanni L, Emmanuel PJ, et al. "A Great Way to Start the Conversation": Evidence for the Use of an Adolescent Mental Health Chatbot Navigator for Youth at Risk of HIV and Other STIs. J Technol Behav Sci. 2023 May 11:1-10. PMID: 37362063. doi: 10.1007/s41347-023-00315-4.

[15] Grové C. Co-developing a Mental Health and Wellbeing Chatbot With and for Young People. Front Psychiatry. 2020;11:606041. PMID: 33597898. doi: 10.3389/fpsyt.2020.606041.

[16] Holt-Quick C, Warren J, Stasiak K, Williams R, Christie G, Hetrick S, et al. A Chatbot Architecture for Promoting Youth Resilience. Healthier Lives, Digitally Enabled: IOS Press; 2021. p. 99-105. doi: 10.3233/SHTI210017

[17] Ludin N, Holt-Quick C, Hopkins S, Stasiak K, Hetrick S, Warren J, et al. A Chatbot to Support Young People During the COVID-19 Pandemic in New Zealand: Evaluation of the Real-World Rollout of an Open Trial. J Med Internet Res. 2022 Nov 04;24(11):e38743. PMID: 36219754. doi: 10.2196/38743.

[18] Høiland CG, Følstad A, Karahasanovic A. Hi, can I help? Exploring how to design a mental health chatbot for youths. Human Technology. 2020;16(2):139-69. doi:10.17011/ht/urn.202008245640

[19] Brandtzæg PB, Skjuve M, Kristoffer Dysthe KK, Følstad A, editors. When the social becomes non-human: young people's perception of social support in chatbots. Proceedings of the 2021 CHI conference on human factors in computing systems; 2021. doi: 10.1145/3411764.3445318

[20] Oliveira ALS, Matos LN, Junior MC, Delabrida ZNC, editors. An Initial Assessment of a Chatbot for Rumination-Focused Cognitive Behavioral Therapy (RFCBT) in College Students. Computational Science and Its Applications–ICCSA 2021: 21st International Conference, Cagliari, Italy, September 13–16, 2021, Proceedings, Part VI 21; 2021: Springer. doi: 10.1007/978-3-030-86979-3_39

[21] Gabrielli S, Rizzi S, Carbone S, Donisi V. A Chatbot-Based Coaching Intervention for Adolescents to Promote Life Skills: Pilot Study. JMIR Hum Factors. 2020 Feb 14;7(1):e16762. PMID: 32130128. doi: 10.2196/16762.

[22] Williams R, Hopkins S, Frampton C, Holt-Quick C, Merry SN, Stasiak K. 21-day stress detox: open trial of a universal well-being chatbot for young adults. Social Sciences. 2021;10(11):416. doi: 10.3390/socsci10110416

[23] Kretzschmar K, Tyroll H, Pavarini G, Manzini A, Singh I, Group NYPsA. Can Your Phone Be Your Therapist? Young People's Ethical Perspectives on the Use of Fully Automated Conversational Agents (Chatbots) in Mental Health Support. Biomed Inform Insights. 2019;11:1178222619829083. PMID: 30858710. doi: 10.1177/1178222619829083.

[24] De Nieva JO, Joaquin JA, Tan CB, Marc Te RK, Ong E, editors. Investigating students’ use of a mental health chatbot to alleviate academic stress. 6th International ACM In-Cooperation HCI and UX Conference; 2020. doi: 10.1145/3431656.3431657

[25] Maenhout L, Peuters C, Cardon G, Compernolle S, Crombez G, DeSmet A. Participatory Development and Pilot Testing of an Adolescent Health Promotion Chatbot. Front Public Health. 2021;9:724779. PMID: 34858919. doi: 10.3389/fpubh.2021.724779.

[26] Crutzen R, Peters GJ, Portugal SD, Fisser EM, Grolleman JJ. An artificially intelligent chat agent that answers adolescents' questions related to sex, drugs, and alcohol: an exploratory study. J Adolesc Health. 2011 May;48(5):514-9. PMID: 21501812. doi: 10.1016/j.jadohealth.2010.09.002.

[27] Greer S, Ramo D, Chang YJ, Fu M, Moskowitz J, Haritatos J. Use of the Chatbot "Vivibot" to Deliver Positive Psychology Skills and Promote Well-Being Among Young People After Cancer Treatment: Randomized Controlled Feasibility Trial. JMIR Mhealth Uhealth. 2019 Oct 31;7(10):e15018. PMID: 31674920. doi: 10.2196/15018.

[28] Huang J, Li Q, Xue Y, Cheng T, Xu S, Jia J, et al., editors. Teenchat: a chatterbot system for sensing and releasing adolescents’ stress. Health Information Science: 4th International Conference, HIS 2015, Melbourne, Australia, May 28-30, 2015, Proceedings 4; 2015: Springer. doi: 10.1007/978-3-319-19156-0_14

[29] Klos MC, Escoredo M, Joerin A, Lemos VN, Rauws M, Bunge EL. Artificial Intelligence-Based Chatbot for Anxiety and Depression in University Students: Pilot Randomized Controlled Trial. JMIR Form Res. 2021 Aug 12;5(8):e20678. PMID: 34092548. doi: 10.2196/20678.

[30] Gaffney H, Mansell W, Edwards R, Wright J. Manage Your Life Online (MYLO): a pilot trial of a conversational computer-based intervention for problem solving in a student sample. Behav Cogn Psychother. 2014 Nov;42(6):731-46. PMID: 23899405. doi: 10.1017/S135246581300060X.

[31] Liu H, Peng H, Song X, Xu C, Zhang M. Using AI chatbots to provide self-help depression interventions for university students: A randomized trial of effectiveness. Internet Interv. 2022 Mar;27:100495. PMID: 35059305. doi: 10.1016/j.invent.2022.100495.

[32] Matheson EL, Smith HG, Amaral ACS, Meireles JFF, Almeida MC, Linardon J, et al. Using Chatbot Technology to Improve Brazilian Adolescents' Body Image and Mental Health at Scale: Randomized Controlled Trial. JMIR Mhealth Uhealth. 2023 Jun 19;11:e39934. PMID: 37335604. doi: 10.2196/39934.

[33] Fabian KE, Foster KT, Chwastiak L, Turner M, Wagenaar BH. Adapting a transdiagnostic digital mental health intervention for use among immigrant and refugee youth in Seattle: a human-centered design approach. Transl Behav Med. 2023 Nov 05;13(11):867-75. PMID: 37418614. doi: 10.1093/tbm/ibad041.

[34] Escobar-Viera CG, Porta G, Coulter RWS, Martina J, Goldbach J, Rollman BL. A chatbot-delivered intervention for optimizing social media use and reducing perceived isolation among rural-living LGBTQ+ youth: Development, acceptability, usability, satisfaction, and utility. Internet Interv. 2023 Dec;34:100668. PMID: 37746640. doi: 10.1016/j.invent.2023.100668.

[35] Viduani A, Cosenza V, Fisher HL, Buchweitz C, Piccin J, Pereira R, et al. Assessing Mood With the Identifying Depression Early in Adolescence Chatbot (IDEABot): Development and Implementation Study. JMIR Hum Factors. 2023 Aug 07;10:e44388. PMID: 37548996. doi: 10.2196/44388.

[36] Wrightson-Hester AR, Anderson G, Dunstan J, McEvoy PM, Sutton CJ, Myers B, et al. An Artificial Therapist (Manage Your Life Online) to Support the Mental Health of Youth: Co-Design and Case Series. JMIR Hum Factors. 2023 Jul 21;10:e46849. PMID: 37477969. doi: 10.2196/46849.

[37] Palma R, Lam HC, Shrivastava A, Karlinsey E, Nguyen K, Deol P, et al., editors. “Monday Feels Like Friday!”-Towards Overcoming Anxiety and Stress of Autistic Young Adults During Times of Isolation. International Conference on Information; 2023: Springer. doi: 10.1007/978-3-031-28032-0_24

[38] Kang A, Hetrick S, Cargo T, Hopkins S, Ludin N, Bodmer S, et al. Exploring Young Adults' Views About Aroha, a Chatbot for Stress Associated With the COVID-19 Pandemic: Interview Study Among Students. JMIR Form Res. 2023 Oct 12;7:e44556. PMID: 37527545. doi: 10.2196/44556.

[39] Afrin Z, Farid DM, Mamun KAA, editors. A Cloud-Based Intelligent Virtual Assistant for Adolescents. International Conference on Intelligent Systems and Data Science; 2023: Springer. doi: 10.1007/978-981-99-7649-2_9
