# Supplementary material for: Current Landscape and Future Directions for Mental Health Conversational Agents for Youth: Scoping Review
Source: JMIR Med Inform. 2025 Feb 28;13:e62758. doi: 10.2196/62758 (PMC11909484; doi:10.2196/62758)
Supplement: Multimedia Appendix 5 [file medinform_v13i1e62758_app5.docx]

**Multimedia Appendix 5. Evaluation outcomes of included studies.**

| **References** | **Dependent Measures** | **Strengths** | **Weaknesses** | **Ethical Considerations** |
| --- | --- | --- | --- | --- |
| Koulouri et al. [1] | Acceptability | Immediate and anytime support, complementing professional services, personalized support, increased awareness | Concerns for data privacy and trust, accuracy of NLP, potential overreliance on chatbot | Data Privacy and Trust |
| Fitzpatrick et al. [2] | Effectiveness, Acceptability, Usability | Effective in reducing depression, CA felt empathetic | Did not specify | Did not specify |
| Kuhlmeier et al. [3] | Personalization | Personalization of avatar, therapeutic content | Insufficient automated adaptation would be worse than a non-adaptive system | Did not specify |
| Elmasri et al. [4] | Usability | Did not specify | Users were frustrated by the need to type their utterances rather than speak naturally, the inability of the chatbot to recognize different keywords that those with which it had been programmed, too much information in chatbot due to structured conversation maps. | Did not specify |
| Abreu et al. [5] | Effectiveness | Effective in tracking screentime | Did not specify | Did not specify |
| Nicol et al. [6] | Effectiveness, Usability, Acceptability | Immediate access to therapy, increased anonymity and reduction in perception of stigma, reduction in time, cost, and efforts for in-person therapy | Potential risks of missing reports of suicidal ideation, overreliance on chatbot, reduced self-efficacy for adaptive help seeking, increased screentime, concerns for privacy and confidentiality of sensitive information | Did not specify |
| Beilharz et al. [7] | Acceptability, Usability | 24/7 availability, engaging, resourceful, nonjudgemental | Not sufficiently capable of understanding users' typed queries | Did not specify |
| Mariamo et al. [8] | Acceptability | Did not specify | Did not specify | Did not specify |
| Dosovitsky et al. [9] | Effectiveness | Availability, effective in symptom improvement, confidentiality of conversation | CAs misunderstand user queries, a lack of personalized answer, content was not appropriate for adolescents | Did not specify |
| Boggiss et al. [10] | Acceptability | Clinical effectiveness, relevant and flexible tools | Did not specify | Did not specify |
| Gabrielli et al. [11] | Effectiveness, Usability | Effective in self-reflection, looks like nice, smart, trustworthy friend | Unclear dialogue, not empathetic, redundancy in content, too much text | Did not specify |
| Schick et al. [12] | Validity/Accuracy, Usability | Did not specify | Did not specify | Did not specify |
| He et al. [13] | Effectiveness, Usability, Acceptability | Effective in reducing depression and emotional relief, convenience, helpful, personalized content | Inflexible and repetitive content/response, technical immaturity | Did not specify |
| Sanabria et al. [14] | Usability, Acceptability | engaging, accessible, useful, confidentiality and privacy | Too fast responses, interface of content with too much information | Privacy and Confidentiality |
| Grové et al. [15] | Usability | Did not specify | Repetitive and limited responses, robotic and unreal interaction | Did not specify |
| Holt-Quick et al. [16] | Usability | Did not specify | Did not specify | Did not specify |
| Ludin et al. [17] | Usability, Acceptability | Accessible, acceptable, engaging, useful, interactivity | Lack of personalized content, limited responses | Did not specify |
| Høiland et al. [18] | Effectiveness, Usability | Accessibility, availability, anonymity, sense of personalized informational support, sense of caring | Limited resources and content, privacy and/or legal issues, navigating and making sense of online information sources is challenging | Did not specify |
| Brandtzæg et al. [19] | Effectiveness | Effective, emotionally supportive, easy to use, availability, quality of information, anonymity, confidentiality | Lack of trust toward non-human, concerns for data privacy | privacy and trust |
| Oliveira et al. [20] | Effectiveness | Did not specify | Did not specify | Did not specify |
| Gabrielli et al. [21] | Effectiveness, User engagement, Usability | Originality of content, human-like interaction, multimedia content, interactivity, availability | Repetitive chatbot questions, lack of personalization | Did not specify |
| Williams et al. [22] | Effectiveness, Acceptability, User engagement, Usability | Informative, engaging, perceived connection/friendship with bot, ease of use, accessibility, interactivity | Lack of personalization, lack of understanding user input, feeling of loneliness or disconnect when talking with bot, repetitive and limited response | Did not specify |
| Kretzschmar et al. [23] | Safety, Privacy and confidentiality | Accessibility, confidentiality, effective, engaging | Too generic content, limited understanding of user input, lack of personalization, lack of empathy | Safety, Privacy, and Confidentiality |
| De Nieva et al. [24] | Effectiveness, Usability | Informative, caring, interactivity, friendly, effective in reducing stress | Ineffective content, inability to understand user input, irrelevant responses, lack of personalization, lengthy textual instruction caused confusion | Did not specify |
| Maenhout et al. [25] | Engagement, Acceptability | Informative, supportive | Redundant responses, technical immaturity, inaccurate or irrelevant responses, too fast responses | Did not specify |
| Crutzen et al. [26] | Engagement, Usability | Did not specify | Did not specify | Did not specify |
| Greer et al. [27] | Engagement, Usability, Effectiveness | Nonjudgemental, helpful, quality of content | Did not specify | Did not specify |
| Huang et al. [28] | Effectiveness, Accuracy | Did not specify | Did not specify | Did not specify |
| Klos et al. [29] | Engagement, Effectiveness | Did not specify | Inaccurate responses | Did not specify |
| Gaffney et al. [30] | Effectiveness, Acceptability | Did not specify | Did not specify | Did not specify |
| Liu et al. [31] | Effectiveness, Usability, User engagement | Did not specify | Did not specify | Did not specify |
| Matheson et al. [32] | Effectiveness, User engagement | Did not specify | Did not specify | Did not specify |
| Fabian et al. [33] | Usability | Friendly and human-like interaction | Repetitive or redundant content | Did not specify |
| Escobar-Viera et al. [34] | Acceptability, Usability, Effectiveness | Interactions, ease of use, interesting features | Chatbot felt robotic and not smart enough, lack of content on other topics important to LGBTQ+ youth, insufficient content on social media interactions and ways of delivering said content | Did not specify |
| Viduani et al. [35] | Acceptability, User engagement | Accessibility, inclusive, easy to use, enjoyable, acceptable | Limited response range, technical susceptibility to changes in the platform, concerns for data privacy, rule-based approach may not be suitable for effectively responding to imminent risks | Did not specify |
| Wrightson-Hester et al. [36] | Acceptability, User engagement, Effectiveness | Acceptable | Limited understanding of user input, hard to express their feelings in text, hard to understand repetition/vagueness of questions CA asked | Did not specify |
| Palma et al. [37] | Acceptability | Novelty, Acceptable | Did not specify | Did not specify |
| Kang et al. [38] | Acceptability, Usability | Friendly and human-like interaction, culturally specific CA character, use of multimedia, accessibility, anonymity, confidentiality, interactivity | Too much text, CAs did not fully understand user input, limited responses, technical bugs, interaction with non-human | Did not specify |
| Afrin et al. [39] | Usability, Acceptability | Did not specify | Did not specify | Did not specify |

References

[1] Koulouri T, Macredie RD, Olakitan D. Chatbots to support young adults’ mental health: An exploratory study of acceptability. ACM Transactions on Interactive Intelligent Systems (TiiS). 2022;12(2):1-39. doi: 10.1145/3485874

[2] Fitzpatrick KK, Darcy A, Vierhile M. Delivering Cognitive Behavior Therapy to Young Adults With Symptoms of Depression and Anxiety Using a Fully Automated Conversational Agent (Woebot): A Randomized Controlled Trial. JMIR Ment Health. 2017 Jun 06;4(2):e19. PMID: 28588005. doi: 10.2196/mental.7785.

[3] Kuhlmeier FO, Gnewuch U, Lüttke S, Brakemeier E-L, Mädche A. A Personalized Conversational Agent to Treat Depression in Youth and Young Adults – A Transdisciplinary Design Science Research Project. Lecture Notes in Computer Science (including subseries Lecture Notes in Artificial Intelligence and Lecture Notes in Bioinformatics); 2022. doi: 10.1007/978-3-031-06516-3_3

[4] Elmasri D, Maeder A, editors. A conversational agent for an online mental health intervention. Brain Informatics and Health: International Conference, BIH 2016, Omaha, NE, USA, October 13-16, 2016 Proceedings; 2016: Springer. doi: 10.1007/978-3-319-47103-7_24

[5] Abreu C, Campos PF, editors. Raising awareness of smartphone overuse among university students: a persuasive systems approach. Informatics; 2022: MDPI. doi: 10.3390/informatics9010015

[6] Nicol G, Wang R, Graham S, Dodd S, Garbutt J. Chatbot-Delivered Cognitive Behavioral Therapy in Adolescents With Depression and Anxiety During the COVID-19 Pandemic: Feasibility and Acceptability Study. JMIR Form Res. 2022 Nov 22;6(11):e40242. PMID: 36413390. doi: 10.2196/40242.

[7] Beilharz F, Sukunesan S, Rossell SL, Kulkarni J, Sharp G. Development of a Positive Body Image Chatbot (KIT) With Young People and Parents/Carers: Qualitative Focus Group Study. J Med Internet Res. 2021 Jun 16;23(6):e27807. PMID: 34132644. doi: 10.2196/27807.

[8] Mariamo A, Temcheff CE, Léger PM, Senecal S, Lau MA. Emotional Reactions and Likelihood of Response to Questions Designed for a Mental Health Chatbot Among Adolescents: Experimental Study. JMIR Hum Factors. 2021 Mar 18;8(1):e24343. PMID: 33734089. doi: 10.2196/24343.

[9] Dosovitsky G, Bunge E. Development of a chatbot for depression: adolescent perceptions and recommendations. Child Adolesc Ment Health. 2023 Feb;28(1):124-7. PMID: 36507594. doi: 10.1111/camh.12627.

[10] Boggiss A, Consedine N, Hopkins S, Silvester C, Jefferies C, Hofman P, et al. Improving the Well-being of Adolescents With Type 1 Diabetes During the COVID-19 Pandemic: Qualitative Study Exploring Acceptability and Clinical Usability of a Self-compassion Chatbot. JMIR Diabetes. 2023 May 05;8:e40641. PMID: 36939680. doi: 10.2196/40641.

[11] Gabrielli S, Rizzi S, Bassi G, Carbone S, Maimone R, Marchesoni M, et al. Engagement and Effectiveness of a Healthy-Coping Intervention via Chatbot for University Students During the COVID-19 Pandemic: Mixed Methods Proof-of-Concept Study. JMIR Mhealth Uhealth. 2021 May 28;9(5):e27965. PMID: 33950849. doi: 10.2196/27965.

[12] Schick A, Feine J, Morana S, Maedche A, Reininghaus U. Validity of Chatbot Use for Mental Health Assessment: Experimental Study. JMIR Mhealth Uhealth. 2022 Oct 31;10(10):e28082. PMID: 36315228. doi: 10.2196/28082.

[13] He Y, Yang L, Zhu X, Wu B, Zhang S, Qian C, et al. Mental Health Chatbot for Young Adults With Depressive Symptoms During the COVID-19 Pandemic: Single-Blind, Three-Arm Randomized Controlled Trial. J Med Internet Res. 2022 Nov 21;24(11):e40719. PMID: 36355633. doi: 10.2196/40719.

[14] Sanabria G, Greene KY, Tran JT, Gilyard S, DiGiovanni L, Emmanuel PJ, et al. "A Great Way to Start the Conversation": Evidence for the Use of an Adolescent Mental Health Chatbot Navigator for Youth at Risk of HIV and Other STIs. J Technol Behav Sci. 2023 May 11:1-10. PMID: 37362063. doi: 10.1007/s41347-023-00315-4.

[15] Grové C. Co-developing a Mental Health and Wellbeing Chatbot With and for Young People. Front Psychiatry. 2020;11:606041. PMID: 33597898. doi: 10.3389/fpsyt.2020.606041.

[16] Holt-Quick C, Warren J, Stasiak K, Williams R, Christie G, Hetrick S, et al. A Chatbot Architecture for Promoting Youth Resilience. Healthier Lives, Digitally Enabled: IOS Press; 2021. p. 99-105. doi: 10.3233/SHTI210017

[17] Ludin N, Holt-Quick C, Hopkins S, Stasiak K, Hetrick S, Warren J, et al. A Chatbot to Support Young People During the COVID-19 Pandemic in New Zealand: Evaluation of the Real-World Rollout of an Open Trial. J Med Internet Res. 2022 Nov 04;24(11):e38743. PMID: 36219754. doi: 10.2196/38743.

[18] Høiland CG, Følstad A, Karahasanovic A. Hi, can I help? Exploring how to design a mental health chatbot for youths. Human Technology. 2020;16(2):139-69. doi:10.17011/ht/urn.202008245640

[19] Brandtzæg PB, Skjuve M, Kristoffer Dysthe KK, Følstad A, editors. When the social becomes non-human: young people's perception of social support in chatbots. Proceedings of the 2021 CHI conference on human factors in computing systems; 2021. doi: 10.1145/3411764.3445318

[20] Oliveira ALS, Matos LN, Junior MC, Delabrida ZNC, editors. An Initial Assessment of a Chatbot for Rumination-Focused Cognitive Behavioral Therapy (RFCBT) in College Students. Computational Science and Its Applications–ICCSA 2021: 21st International Conference, Cagliari, Italy, September 13–16, 2021, Proceedings, Part VI 21; 2021: Springer. doi: 10.1007/978-3-030-86979-3_39

[21] Gabrielli S, Rizzi S, Carbone S, Donisi V. A Chatbot-Based Coaching Intervention for Adolescents to Promote Life Skills: Pilot Study. JMIR Hum Factors. 2020 Feb 14;7(1):e16762. PMID: 32130128. doi: 10.2196/16762.

[22] Williams R, Hopkins S, Frampton C, Holt-Quick C, Merry SN, Stasiak K. 21-day stress detox: open trial of a universal well-being chatbot for young adults. Social Sciences. 2021;10(11):416. doi: 10.3390/socsci10110416

[23] Kretzschmar K, Tyroll H, Pavarini G, Manzini A, Singh I, Group NYPsA. Can Your Phone Be Your Therapist? Young People's Ethical Perspectives on the Use of Fully Automated Conversational Agents (Chatbots) in Mental Health Support. Biomed Inform Insights. 2019;11:1178222619829083. PMID: 30858710. doi: 10.1177/1178222619829083.

[24] De Nieva JO, Joaquin JA, Tan CB, Marc Te RK, Ong E, editors. Investigating students’ use of a mental health chatbot to alleviate academic stress. 6th International ACM In-Cooperation HCI and UX Conference; 2020. doi: 10.1145/3431656.3431657

[25] Maenhout L, Peuters C, Cardon G, Compernolle S, Crombez G, DeSmet A. Participatory Development and Pilot Testing of an Adolescent Health Promotion Chatbot. Front Public Health. 2021;9:724779. PMID: 34858919. doi: 10.3389/fpubh.2021.724779.

[26] Crutzen R, Peters GJ, Portugal SD, Fisser EM, Grolleman JJ. An artificially intelligent chat agent that answers adolescents' questions related to sex, drugs, and alcohol: an exploratory study. J Adolesc Health. 2011 May;48(5):514-9. PMID: 21501812. doi: 10.1016/j.jadohealth.2010.09.002.

[27] Greer S, Ramo D, Chang YJ, Fu M, Moskowitz J, Haritatos J. Use of the Chatbot "Vivibot" to Deliver Positive Psychology Skills and Promote Well-Being Among Young People After Cancer Treatment: Randomized Controlled Feasibility Trial. JMIR Mhealth Uhealth. 2019 Oct 31;7(10):e15018. PMID: 31674920. doi: 10.2196/15018.

[28] Huang J, Li Q, Xue Y, Cheng T, Xu S, Jia J, et al., editors. Teenchat: a chatterbot system for sensing and releasing adolescents’ stress. Health Information Science: 4th International Conference, HIS 2015, Melbourne, Australia, May 28-30, 2015, Proceedings 4; 2015: Springer. doi: 10.1007/978-3-319-19156-0_14

[29] Klos MC, Escoredo M, Joerin A, Lemos VN, Rauws M, Bunge EL. Artificial Intelligence-Based Chatbot for Anxiety and Depression in University Students: Pilot Randomized Controlled Trial. JMIR Form Res. 2021 Aug 12;5(8):e20678. PMID: 34092548. doi: 10.2196/20678.

[30] Gaffney H, Mansell W, Edwards R, Wright J. Manage Your Life Online (MYLO): a pilot trial of a conversational computer-based intervention for problem solving in a student sample. Behav Cogn Psychother. 2014 Nov;42(6):731-46. PMID: 23899405. doi: 10.1017/S135246581300060X.

[31] Liu H, Peng H, Song X, Xu C, Zhang M. Using AI chatbots to provide self-help depression interventions for university students: A randomized trial of effectiveness. Internet Interv. 2022 Mar;27:100495. PMID: 35059305. doi: 10.1016/j.invent.2022.100495.

[32] Matheson EL, Smith HG, Amaral ACS, Meireles JFF, Almeida MC, Linardon J, et al. Using Chatbot Technology to Improve Brazilian Adolescents' Body Image and Mental Health at Scale: Randomized Controlled Trial. JMIR Mhealth Uhealth. 2023 Jun 19;11:e39934. PMID: 37335604. doi: 10.2196/39934.

[33] Fabian KE, Foster KT, Chwastiak L, Turner M, Wagenaar BH. Adapting a transdiagnostic digital mental health intervention for use among immigrant and refugee youth in Seattle: a human-centered design approach. Transl Behav Med. 2023 Nov 05;13(11):867-75. PMID: 37418614. doi: 10.1093/tbm/ibad041.

[34] Escobar-Viera CG, Porta G, Coulter RWS, Martina J, Goldbach J, Rollman BL. A chatbot-delivered intervention for optimizing social media use and reducing perceived isolation among rural-living LGBTQ+ youth: Development, acceptability, usability, satisfaction, and utility. Internet Interv. 2023 Dec;34:100668. PMID: 37746640. doi: 10.1016/j.invent.2023.100668.

[35] Viduani A, Cosenza V, Fisher HL, Buchweitz C, Piccin J, Pereira R, et al. Assessing Mood With the Identifying Depression Early in Adolescence Chatbot (IDEABot): Development and Implementation Study. JMIR Hum Factors. 2023 Aug 07;10:e44388. PMID: 37548996. doi: 10.2196/44388.

[36] Wrightson-Hester AR, Anderson G, Dunstan J, McEvoy PM, Sutton CJ, Myers B, et al. An Artificial Therapist (Manage Your Life Online) to Support the Mental Health of Youth: Co-Design and Case Series. JMIR Hum Factors. 2023 Jul 21;10:e46849. PMID: 37477969. doi: 10.2196/46849.

[37] Palma R, Lam HC, Shrivastava A, Karlinsey E, Nguyen K, Deol P, et al., editors. “Monday Feels Like Friday!”-Towards Overcoming Anxiety and Stress of Autistic Young Adults During Times of Isolation. International Conference on Information; 2023: Springer. doi: 10.1007/978-3-031-28032-0_24

[38] Kang A, Hetrick S, Cargo T, Hopkins S, Ludin N, Bodmer S, et al. Exploring Young Adults' Views About Aroha, a Chatbot for Stress Associated With the COVID-19 Pandemic: Interview Study Among Students. JMIR Form Res. 2023 Oct 12;7:e44556. PMID: 37527545. doi: 10.2196/44556.

[39] Afrin Z, Farid DM, Mamun KAA, editors. A Cloud-Based Intelligent Virtual Assistant for Adolescents. International Conference on Intelligent Systems and Data Science; 2023: Springer. doi: 10.1007/978-981-99-7649-2_9
